# Supplementary material for: miR-151-5p regulates neural stem cell fate by targeting APH1A to modulate Notch signaling gradients
Source: Stem Cell Reports. 2026 May 21;21(6):102927. doi: 10.1016/j.stemcr.2026.102927 (PMC13261887; doi:10.1016/j.stemcr.2026.102927)
Supplement: Document S1. Figures S1–S5 [file mmc1.pdf]

Stem Cell Reports, Volume *21*

Supplemental Information

miR-151-5p regulates neural stem cell fate by targeting APH1A to modulate Notch signaling gradients

Xinrun Wang, Li Li, Zhuo Chen, Yi Zeng, Pengcheng Shu, Lin Hou, Bin Yin, Wei Liu, and Xiaozhong Peng

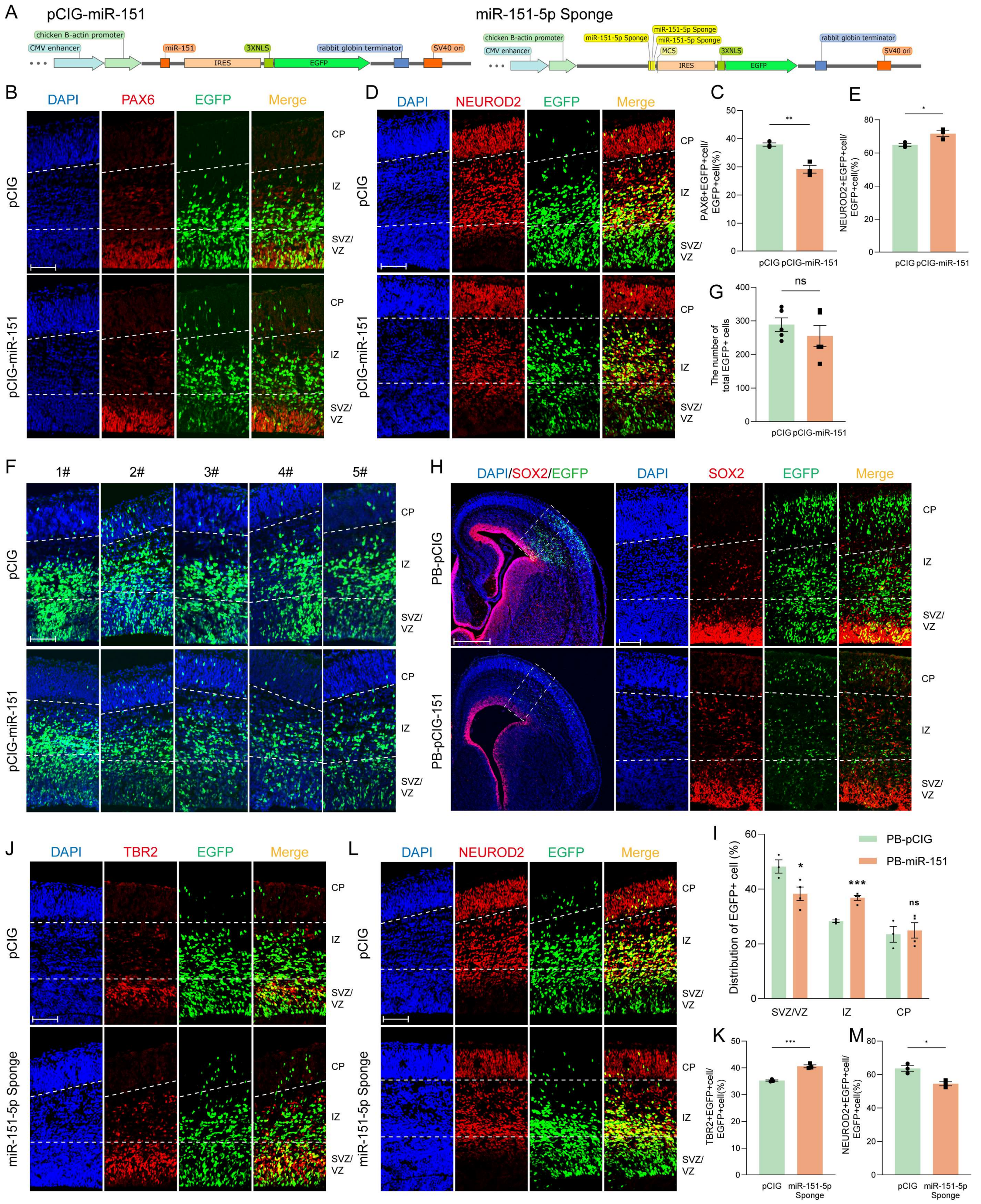

Supplementary Figure 1. miR-151-5p influences the outward migration of NSCs.

(A) Schematic diagram showing pCIG-miR-151 (overexpression) and miR-151-5p Sponge (knockdown) plasmid.

(B) Immunostaining of E15.5 brain sections electroporated with control (pCIG) or OE (pCIG-miR-151) on E13.5. White-dotted rectangles mark comparable regions for magnification.

(C) Quantification of the ratio of PAX6^{+}EGFP^{+}cells in all EGFP^{+} cells.

(D) Immunostaining of E15.5 brain sections electroporated with control (pCIG) or OE (pCIG-miR-151) on E13.5. White-dotted rectangles mark comparable regions for magnification.

(E) Quantification of the ratio of NEUROD2^{+}EGFP^{+}cells in all EGFP^{+} cells.

(F) Representative images from five independent experiments, showing E15.5 brain sections that were electroporated on E13.5 with the control (pCIG) or OE (pCIG-miR-151).

(G) Quantitative analysis of the number of total EGFP^{+} cells, n=5 biological replicates.

(H) Immunostaining of E16.5 brain sections electroporated with control (PB-pCIG) or miR-151-OE plasmid on E13.5. White-dotted rectangles mark comparable regions for magnification.

(I) Quantitative analysis of the distribution of EGFP^{+} cells after dividing the neocortex into 3 parts (SVZ/VZ, IZ, CP) (n=3 for the control group, n=4 for the miR-151 OE group).

(J) Immunostaining of E15.5 brain sections electroporated with control (pCIG) or Down (miR-151-5p Sponge) on E13.5. White-dotted rectangles mark comparable regions for magnification.

(K) Quantification of the ratio of TBR2^{+}EGFP^{+}cells in all EGFP^{+} cells.

(L) Immunostaining of E15.5 brain sections electroporated with control (pCIG) or Down (miR-151-5p Sponge) on E13.5. White-dotted rectangles mark comparable regions for magnification.

(M) Quantification of the ratio of NEUROD2^{+}EGFP^{+}cells in all EGFP^{+} cells.

Scale bars for the 10x images, 500 μm; 40x images, 100 μm. Data are presented as mean ± SEM. Individual data points represent independent biological replicates. Statistical analysis was performed by unpaired 2-tailed Student's *t* test; ns, not significant; * $p < 0.05$; *** $p < 0.001$.

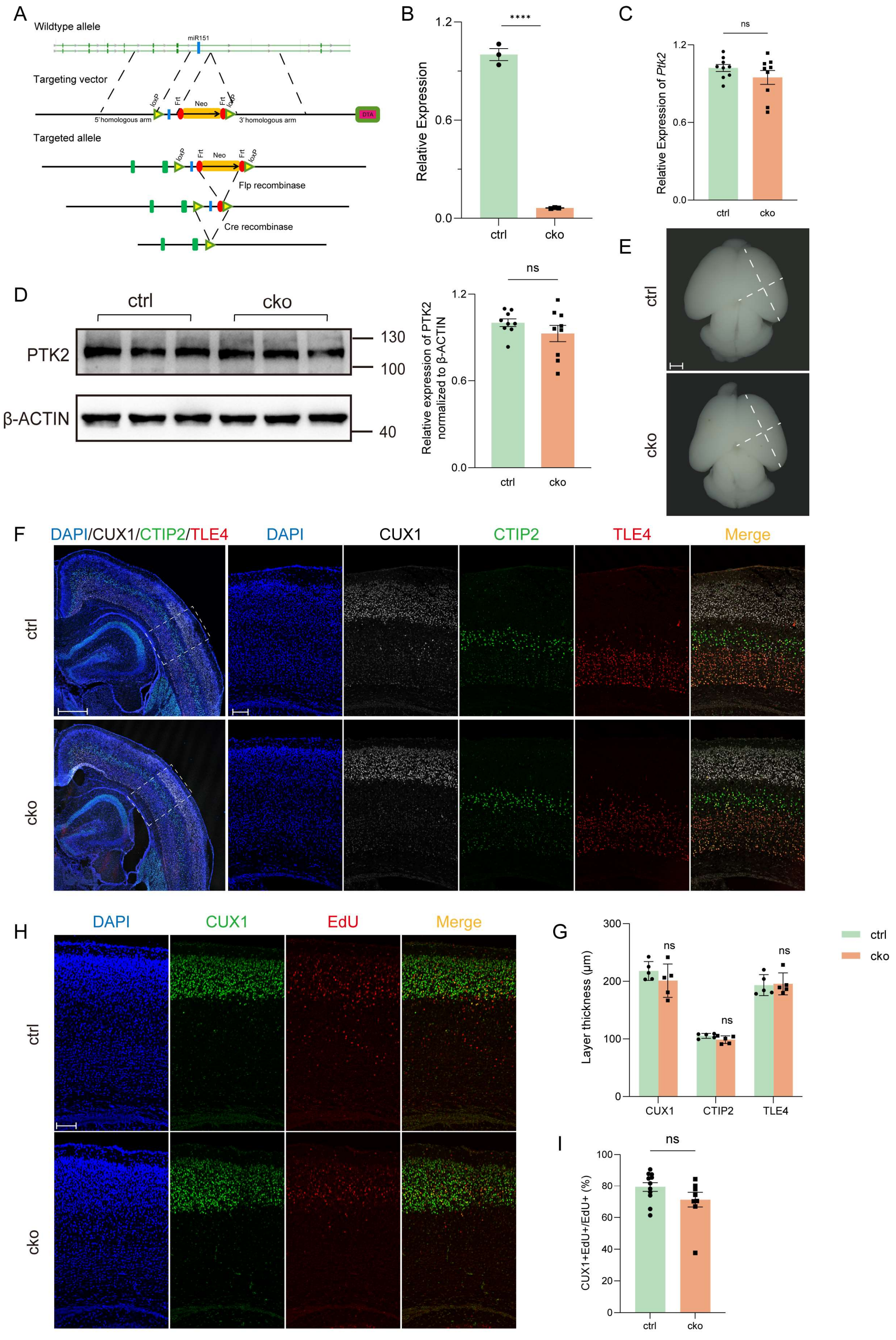

Supplementary Figure 2. Knockout miR-151-5p does not affect the construct of the cerebral cortex in postnatal mice.

(A) Schematic diagram of the construction strategy of conditional knockout miR-151 mouse, loxP was inserted on the intron sequence between exons 22 and 23 of *Ptk2* gene.

(B) RT-qPCR measurement of miR-151-5p expression level in E14.5 miR-151 cko and ctrl NSC, n=3 biological replicates.

(C) Measurement of *Ptk2* mRNA expression in NSCs derived from E14.5 ctrl and miR-151-cko littermate mice. n=3 biological replicates.

(D) Western blot of PTK2 in NSCs from E14.5 miR-151 cko and ctrl littermate mice. β-ACTIN is used as a loading control. ImageJ is used to analyze the gray scale of signals.

(E) Brain dissection revealed that loss of miR-151 did not affect the dorsal forebrain at P3 mouse. Scale bar, 1mm.

(F) Immunostaining of P3 brain sections derived from miR-151 cko and ctrl littermate mice. Sections were stained with DAPI and various markers (CUX1, CTIP2, TLE4). White-dotted rectangles mark comparable regions for magnification.

(G) Quantitative analysis of the thickness of different cortical layers, n=5 biological replicates.

(H) Immunostaining of P3 brain sections derived from miR-151 cko and ctrl littermate mice. Sections were stained with DAPI, upper-layer marker CUX1 and EdU labeling.

(I) Quantitative analysis of the ratio of $CUX1^{+}EdU^{+}$ cells in all EdU^{+} cells, n=7 biological replicates.

Scale bars for the 10x images, 500 μm; 40x images, 100 μm. Data are presented as mean ± SEM. Individual data points represent independent biological replicates. Statistical analysis was performed by unpaired 2-tailed Student's *t* test; ns. not significant; **** $p < 0.0001$.

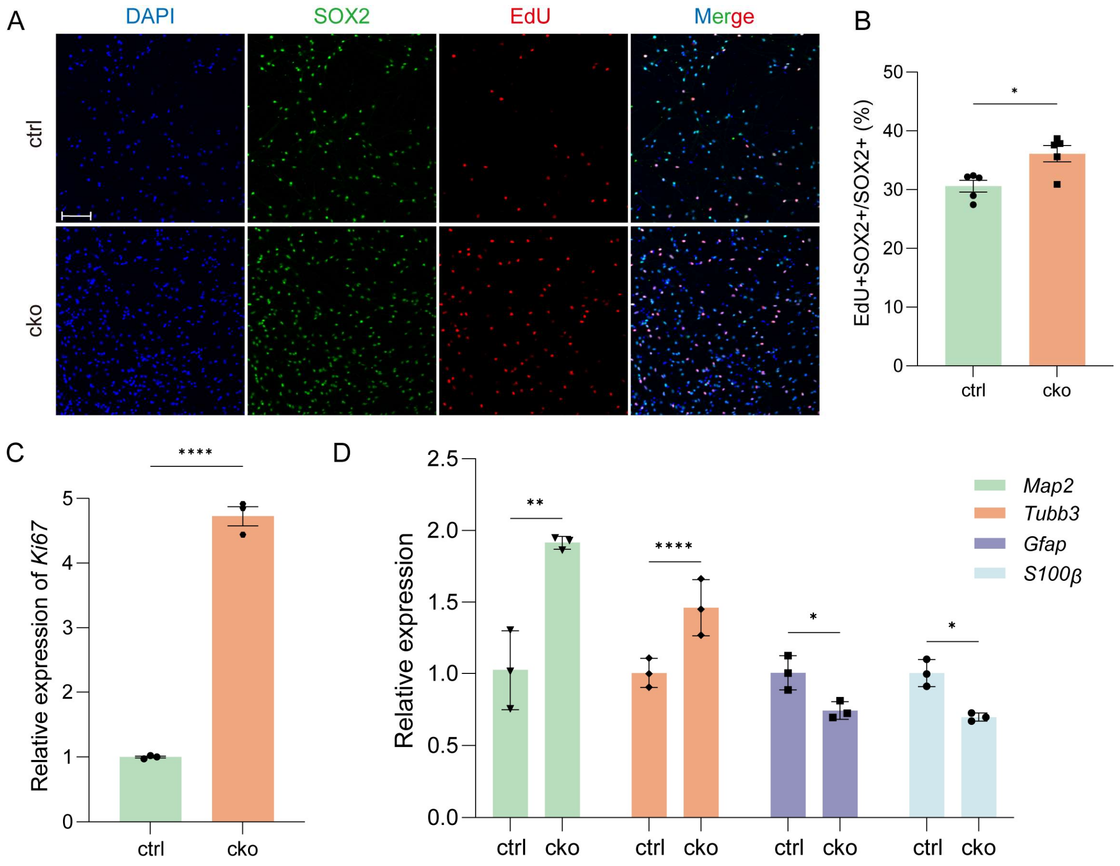

Supplementary Figure 3. Knockout miR-151 changes the proliferative ability and differentiation potential of NSC.

(A) Detection of NSC proliferative ability from E16.5 miR-151 cko and ctrl littermate mice by immunostaining. Scale bar, 100 μm.

(B) Quantitative analysis of the ratio of EdU^+SOX2^+ cells in all $SOX2^+$ cells, n=5 biological replicates.

(C) Measurement of *Ki67* mRNA expression in NSCs derived from E14.5 ctrl and miR-151-cko littermate mice. n=3 biological replicates.

(D) Measurements of neuron markers (*Map2*, *Tubb3*) and astrocyte markers (*Gfap*, *S100β*) mRNA expression in NSCs derived from E16.5 ctrl and miR-151-cko littermate mice. n=3 biological replicates.

Data are presented as mean ± SEM. Individual data points represent independent biological

replicates. Statistical analysis was performed by unpaired 2-tailed Student's *t* test; * $p < 0.05$; ** $p < 0.01$; *** $p < 0.001$; **** $p < 0.0001$.

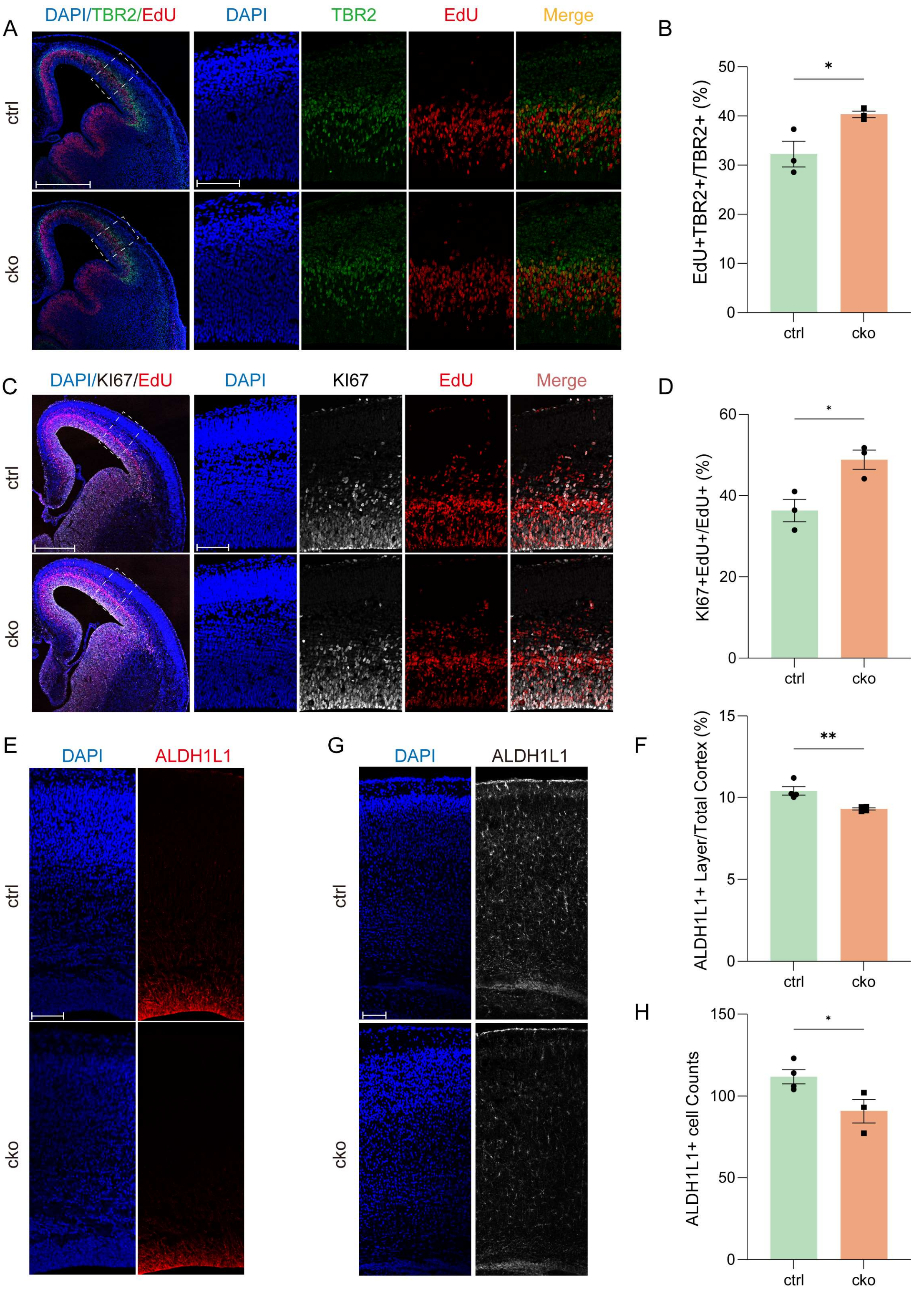

Supplementary Figure 4. Knockout miR-151 decreases ALDH1L1^{+} cells in gliogenesis.

(A) Immunostaining of E13.5 brain sections derived from miR-151 cko and ctrl littermate mice. White-dotted rectangles mark comparable regions for magnification.

(B) Quantitative analysis of the ratio of EdU^{+}TBR2^{+} cells in all TBR2^{+} cells, n=3 biological

replicates.

(C) Immunostaining of E15.5 coronal sections derived from miR-151 cko and ctrl littermate mice. White-dotted rectangles mark comparable regions for magnification.

(D) Quantification of the ratio of $KI67^{+}EdU^{+}$ cells in all EdU^{+} cells.

(E) Immunostaining of E18.5 coronal sections derived from miR-151 cko and ctrl littermate mice. Sections were stained with DAPI staining and astrocyte marker ALDH1L1.

(F) Quantification of the ratio of $ALDH1L1^{+}$ layer in total cortex.

(G) Immunostaining of P3 coronal sections derived from miR-151 cko and ctrl littermate mice. Sections were stained with DAPI staining and astrocyte marker ALDH1L1.

(H) Quantification of the number of $ALDH1L1^{+}$ cells.

Scale bars for the 10x images, 500 μm; 40x images, 100 μm. Data are presented as mean ± SEM. Individual data points represent independent biological replicates. Statistical analysis was performed by unpaired 2-tailed Student's *t* test, n=3 biological replicates; $*p < 0.05$; $**p < 0.01$.

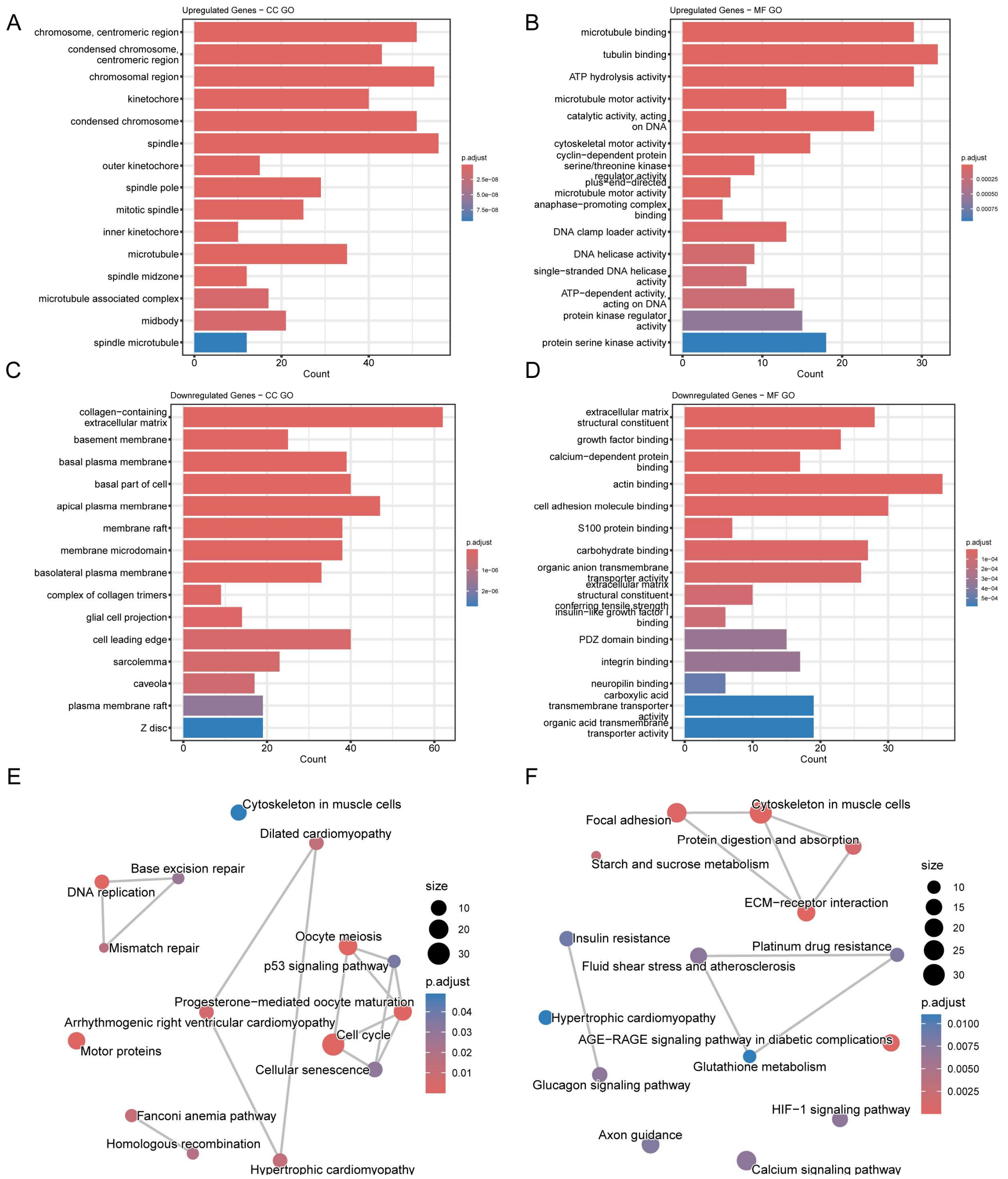

Supplementary Figure 5. GO enrichment results and KEGG-Pathway enrichment results in E14.5 miR-151 cko and ctrl NSCs.

(A) The GO enrichment results of cellular components were obtained by using up-regulated genes.

(B) The GO enrichment results of molecular functions were obtained by using up-regulated genes.

(C) The GO enrichment results of cellular components were obtained by using down-regulated genes.

(D) The GO enrichment results of molecular functions were obtained by using down-regulated genes.

(E) The enrichment map is used to visualize the correlations between various pathways in the results of KEGG-Pathway enrichment analysis by using up-regulated genes.

(F) The enrichment map is used to visualize the correlations between various pathways in the results of KEGG-Pathway enrichment analysis by using down-regulated genes.

All results were analyzed by using clusterProfiler.
