# Supplementary material for: miR-151-5p regulates neural stem cell fate by targeting APH1A to modulate Notch signaling gradients
Source: Stem Cell Reports. 2026 May 21;21(6):102927. doi: 10.1016/j.stemcr.2026.102927 (PMC13261887; doi:10.1016/j.stemcr.2026.102927)
Supplement: Table S1. Primer list [file mmc2.pdf]

Table S1 Primer List

Primer List	Sequence (5’——3’)
Plasmid primer	Sequence (5’——3’)
pCIG-miR-151 F	AAGCTTGATATCGAATTCCCAATGGTGAAGTCCAACTCC
pCIG-miR-151 R	CCCGGGCTGCAGGAATTCTGTAGCTCTCTTGGGTTAGGC
miR-151-5p Sponge F	ATAAGAATGCGGCCGCCATGGACGAGCTGTACAAG
miR-151-5p Sponge R	CCATAATTTTTGGCAGAGG
pCIG-Aph1a-F	TTTGGCAAAGAATTGCTCGAGATGGGGGCTGCTGTGTTTTTC
pCIG-Aph1a-R	CGGGCTGCAGGAATTCTCAGTCCTCGGGTGGGATGC
pCBFRE-mcherry-F	GGCTTTTGCAAAAAGCTTATGGTGAGCAAGGGCGAGG
pCBFRE-mcherry-R	CGGGGGCCACCTGATATCTCACTCGAGCTTGTACAGCTCGT
qPCR primer	Sequence (5’——3’)
Ki67-F	ATCATTGACCGCTCCTTTAGGT
Ki67-R	GCTCGCCTTGATGGTTCCT
Map2-F	GGTCACAGGGCACCTATTCA
Map2-R	TGTTCACCTTTCAGGACTGC
Tubb3-F	TAGACCCCAGCGGCAACTAT
Tubb3-R	GTTCCAGGTTCCAAGTCCACC
Gfap-F	ACCAGCTTACGGCCAACAG
Gfap-R	CCAGCGATTCAACCTTTCTCT
S100β-F	TGGTTGCCCTCATTGATGTCT
S100β-R	CCCATCCCCATCTTCGTCC
Ptk2-F	CCTGCTATGGATTTCGCCTCAG
Ptk2-R	TCCACTCCTCTGGTGGGTG
Gapdh-F	GTGTTCCTACCCCCAATGTGT
Gapdh-R	ATTGTCATACCAGGAAATGAGCTT
