# Supplementary material for: miR-151-5p regulates neural stem cell fate by targeting APH1A to modulate Notch signaling gradients
Source: Stem Cell Reports. 2026 May 21;21(6):102927. doi: 10.1016/j.stemcr.2026.102927 (PMC13261887; doi:10.1016/j.stemcr.2026.102927)
Supplement: Document S2. Article plus supplemental information [file mmc3.pdf]

STEM CELL REPORTS
Article
OPEN ACCESS

miR-151-5p regulates neural stem cell fate by targeting APH1A to modulate Notch signaling gradients

Xinrun Wang,[1,3] Li Li,[2,3] Zhuo Chen,[1] Yi Zeng,[1] Pengcheng Shu,[1] Lin Hou,[1] Bin Yin,[1] Wei Liu,[1,*] and Xiaozhong Peng[1,2,4,*]

[1]State Key Laboratory of Common Mechanism Research for Major Diseases, Department of Biochemistry & Molecular Biology, Medical Primate Research Center, Neuroscience Center, Institute of Basic Medical Sciences Chinese Academy of Medical Sciences, School of Basic Medicine Peking Union Medical College, Beijing 100005, China

[2]State Key Laboratory of Respiratory Health and Multimorbidity, National Center of Technology Innovation for Animal Model, National Human Diseases Animal Model Resource Center, Beijing Engineering Research Center for Experimental Animal Models of Human Critical Diseases, Institute of Laboratory Animal Sciences, CAMS & PUMC, Beijing 100021, China

[3]These authors contributed equally

[4]Lead contact

*Correspondence: liuwei@ibms.cams.cn (W.L.), pengxiaozhong@pumc.edu.cn (X.P.)

https://doi.org/10.1016/j.stemcr.2026.102927

SUMMARY

The precise regulation of neural stem cell (NSC) fate is fundamental to neocortical development. MicroRNAs (miRNAs) are critical post-transcriptional regulators in this process, yet the functions of many remain unknown. Here, we found miR-151-5p is expressed in NSCs of the developing mouse cerebral cortex. Conditional knockout of miR-151-5p increased SOX2 expression in NSCs and enhanced their proliferative capacity. Mechanistically, we identified APH1A, a core subunit of the γ-secretase complex, as a direct target of miR-151-5p. Notably, overexpression of APH1A phenocopied the effects of miR-151-5p knockout, promoting NSC proliferation by elevating NICD levels. These findings demonstrate that miR-151-5p biases NSC fate specification by targeting APH1A to modulate the Notch signaling pathway, thereby fine-tuning the balance between NSC maintenance and differentiation. In summary, our study unveils a novel miR-151-5p/APH1A/Notch signaling axis that governs NSC fate, adding a critical layer of post-transcriptional regulation to our understanding of mammalian neocortical development.

INTRODUCTION

The mammalian neocortex possesses a complex and highly organized six-layered structure, formed by the precise arrangement of diverse neuronal subtypes (Jorstad et al., 2023; Lewis et al., 2021; Wang et al., 2025). The establishment of this intricate architecture is contingent upon the strict regulation of key biological events during cortical development, including neural stem cell (NSC) proliferation, neuronal migration, and differentiation (Götz and Huttner, 2005; Kalogeropoulou et al., 2019). Central to this process is the dynamic regulation of NSCs, which exhibit the capacity for both self-renewing symmetric divisions to maintain the progenitor pool, and asymmetric divisions to generate neurons and intermediate progenitors (Gal et al., 2006; Hippenmeyer, 2023; Kirwan et al., 2015; Rosebrock et al., 2022; Yale et al., 2023).

The stemness maintenance of NSCs is regulated by multiple molecular mechanisms, including core transcription factors (SOX2, PAX6, and HES5), epigenetic regulators (PRCs, MLL family, histone deacetylases, DNA methylation/demethylation, and non-coding RNAs), and the Notch signaling pathway (NOTCH receptors, DELTA ligands, and NUMB) (Fenoglio et al., 2013; Ma et al., 2023; Masui et al., 2007; Mohamed Ariff et al., 2012; Sansom et al., 2009; Shimojo et al., 2008). Among these, the Notch signaling pathway plays a central and indispensable role in maintaining the stemness of NSCs. It ensures the stability of the NSC pool and their self-renewal capacity by inhibiting the premature differentiation into neurons and glial cells (Fiddes et al., 2018; McLaren and Butts, 2025). Within the VZ, Notch activity forms a gradient that is high apically and low basally. High apical Notch signaling maintains the proliferative, undifferentiated state of NSCs, whereas exposure to lower basal Notch signaling prompts cell cycle exit and neuronal differentiation (Del Bene et al., 2008; Liu et al., 2023). This interplay forms a spatiotemporal regulatory loop crucial for balancing proliferation and differentiation.

Within this complex regulatory network, microRNAs (miRNAs) have emerged as pivotal post-transcriptional regulators (Di Bella et al., 2021; Telley et al., 2019). As small, 19–22 nucleotide non-coding RNAs, miRNAs control gene expression by mediating mRNA degradation or translational inhibition (Bartel, 2009). Moreover, they represent a critical layer of regulatory precision ensuring the proper orchestration of NSC fate determination, cell cycle progression, and neuronal migration (Ma et al., 2019; Sun and Shi, 2015; Yapijakis, 2020).

A previous screen from our laboratory examining miRNA expression profiles during mouse cortical development identified that miR-151-5p is localized to NSC in the ventricular zone (VZ), prompting further investigation (Shu et al.,

© 2026 The Authors.
Published by Elsevier Inc. on behalf of International Society for Stem Cell Research.
This is an open access article under the CC BY license (http://creativecommons.org/licenses/by/4.0/).

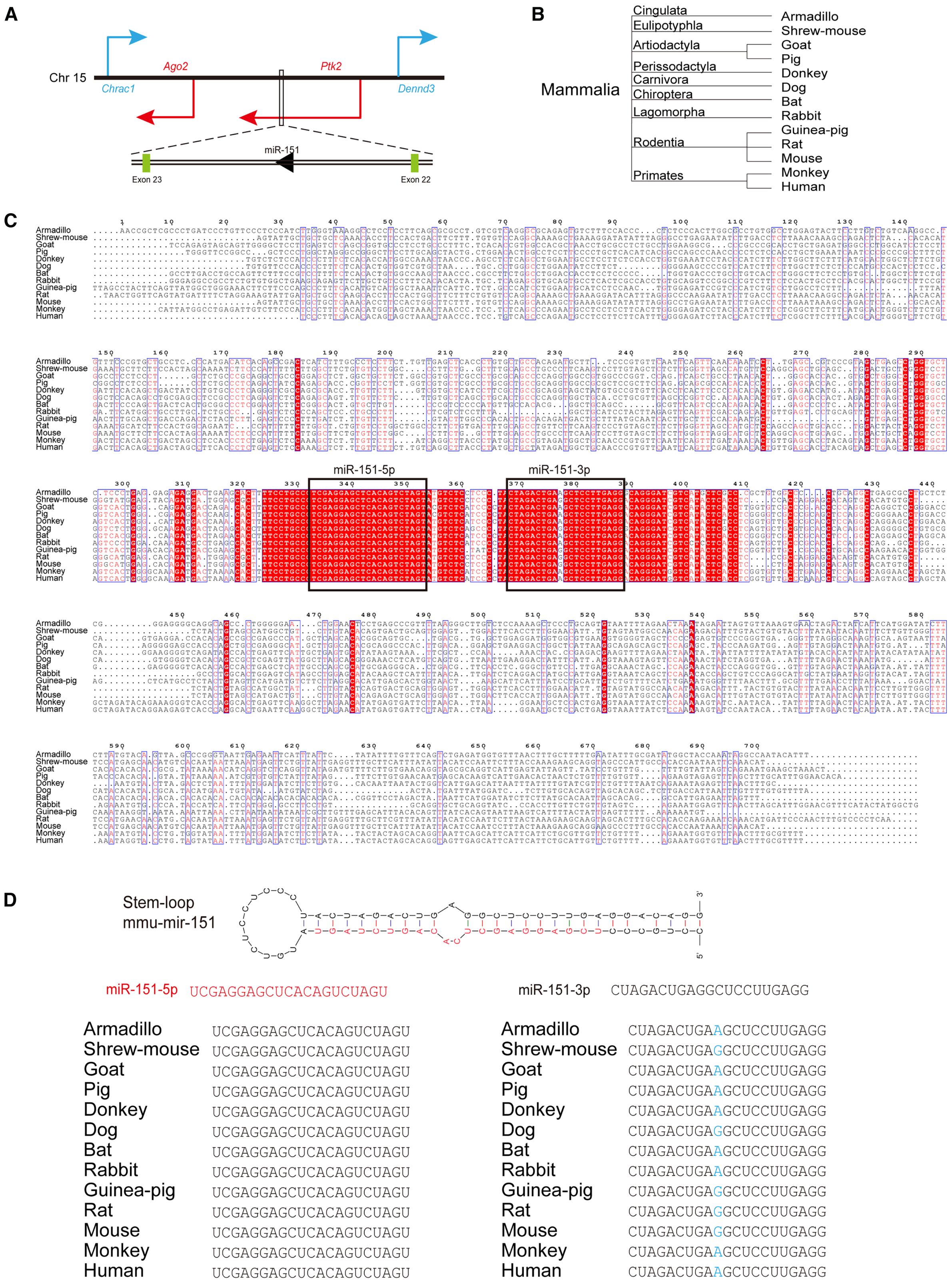

(legend on next page)

2019a). While miR-151-5p has been implicated in various tumors and non-tumor diseases, its role in neurodevelopment remained uncharacterized (Chen et al., 2017; Ding et al., 2010; Huang et al., 2024). Here, we show that miR-151-5p is expressed in NSCs and regulates their proliferation and differentiation. Mechanistically, we identify APH1A, a core subunit of the γ-secretase complex, as a direct target of miR-151-5p. APH1A is crucial for maintaining γ-secretase complex stability and ensuring the correct folding and localization of Presenilin (Sun et al., 2015; Tolia and De Strooper, 2009; Yang et al., 2021). Our findings reveal that by targeting APH1A, miR-151-5p modulates Notch signaling, thereby influencing the balance between NSC proliferation and differentiation during cortical development.

In summary, our research not only elucidates the potential functional mechanism of miR-151-5p in maintaining NSC stemness and regulating differentiation, but also provides crucial evidence for exploring novel regulatory pathways governing the Notch signaling pathway and the cell cycle coordination in the mammalian neocortex.

RESULTS

The sequence of miR-151-5p is highly conserved across species

Our previous screen of miRNAs during mouse cortical development detected miR-151-5p expression in VZ NSCs. Analysis of NCBI and Ensembl databases localized the miR-151 locus within the intron spanning exons 22–23 of the *Ptk2* gene (Figure 1A). In addition, we found miR-151 is expressed exclusively in Mammalia. By categorizing and summarizing all species in the database, we selected 13 species from 9 different orders for sequence alignment analysis (Figure 1B). The results showed that miR-151 exhibits high evolutionary conservation across all mammals. However, the 600 bp region (300 bp upstream and 300 bp downstream) within the intron of its host gene *Ptk2* is not conserved, suggesting the specific functional importance of miR-151 (Figure 1C). The miR-151 precursor folds into a stem-loop structure, which undergoes Dicer-mediated cleavage to yield two mature miRNAs: miR-151-5p (from the 5′ arm) and miR-151-3p (from the 3′ arm). Sequence comparison demonstrated perfect conservation of miR-151-5p across all examined species, whereas miR-151-3p exhibited a single-nucleotide substitution (Figure 1D). This evolutionary constraint implies essential biological roles for miR-151-5p in neural development.

miR-151-5p modulates NSC migration without altering cortical lamination

To further investigate miR-151-5p's function in neural development, we utilized the pCIG vector to construct miR-151 overexpression and knockdown plasmids (Figure S1A). Given that *in utero* electroporation (IUE) is widely adopted in neural development studies for ectopic expression, we performed short-term IUE (E13.5-E15.5) to assess the effects of *in vivo* miR-151–5p overexpression. In the miR-151-5p overexpression group, anatomical quantification revealed a significant shift in cell distribution: the proportion of $EGFP^+$ cells was reduced in the Ventricular/Subventricular Zone (VZ/SVZ) but significantly increased in the Intermediate Zone (IZ) compared to controls (Figures 2A and 2B). To determine whether this phenotype resulted from altered migration kinetics or changes in cell fate, we performed immunostaining for the progenitor markers PAX6 and TBR2, and the neuronal marker NEUROD2. Quantitative analysis showed that miR-151-5p overexpression significantly decreased the proportion of $EGFP^+$ cells co-expressing PAX6 or TBR2, while increasing the fraction of NEUROD2+ neurons (Figures 2C and S1B–S1E). This suggests that the depletion of cells from the VZ/SVZ and their accumulation in the IZ is driven by premature neurogenesis, where progenitors exit the cell cycle early and differentiate into neurons. Meanwhile, the total number of $EGFP^+$ cells showed no significant difference between the overexpression and control groups (Figures S1F and S1G), ruling out overt cell death or massive proliferation defects as the primary cause of this phenotype. Consistent results were obtained using a genomically integrable PB-pCIG-151 plasmid in E13.5-E16.5 IUE experiments (Figures S1H and S1I).

We next constructed a miR-151-5p Sponge plasmid (pCIG-based) and delivered it via E13.5–15.5 IUE to

Figure 1. The sequence of miR-151-5p is highly conserved across species

(A) The relative positions of mmu-miR-151 in the *Chrac1-Dennd3* region are shown. Blue genes (*Chrac1* and *Dennd3*) indicate forward transcription, and red genes (*Ago2* and *Ptk2*) indicate reverse transcription. mmu-miR-151 is in the intron sequence between exons 22 and 23 of the *Ptk2* gene.

(B) The phylogenetic analysis of species expressing miR-151. Summary of miR-151-expressing species from the Ensembl database revealed its exclusive expression in mammals, encompassing nine orders. Thirteen species from these orders were selected for sequence alignment analysis.

(C) Comparative analysis across species revealed that the mature miR-151 sequence was conserved, whereas the flanking 600 bp region in the *Ptk2* intron was not.

(D) The sequence alignment reveals that miR-151-5p is perfectly conserved across species, while miR-151-3p shows only a single-nucleotide variation.

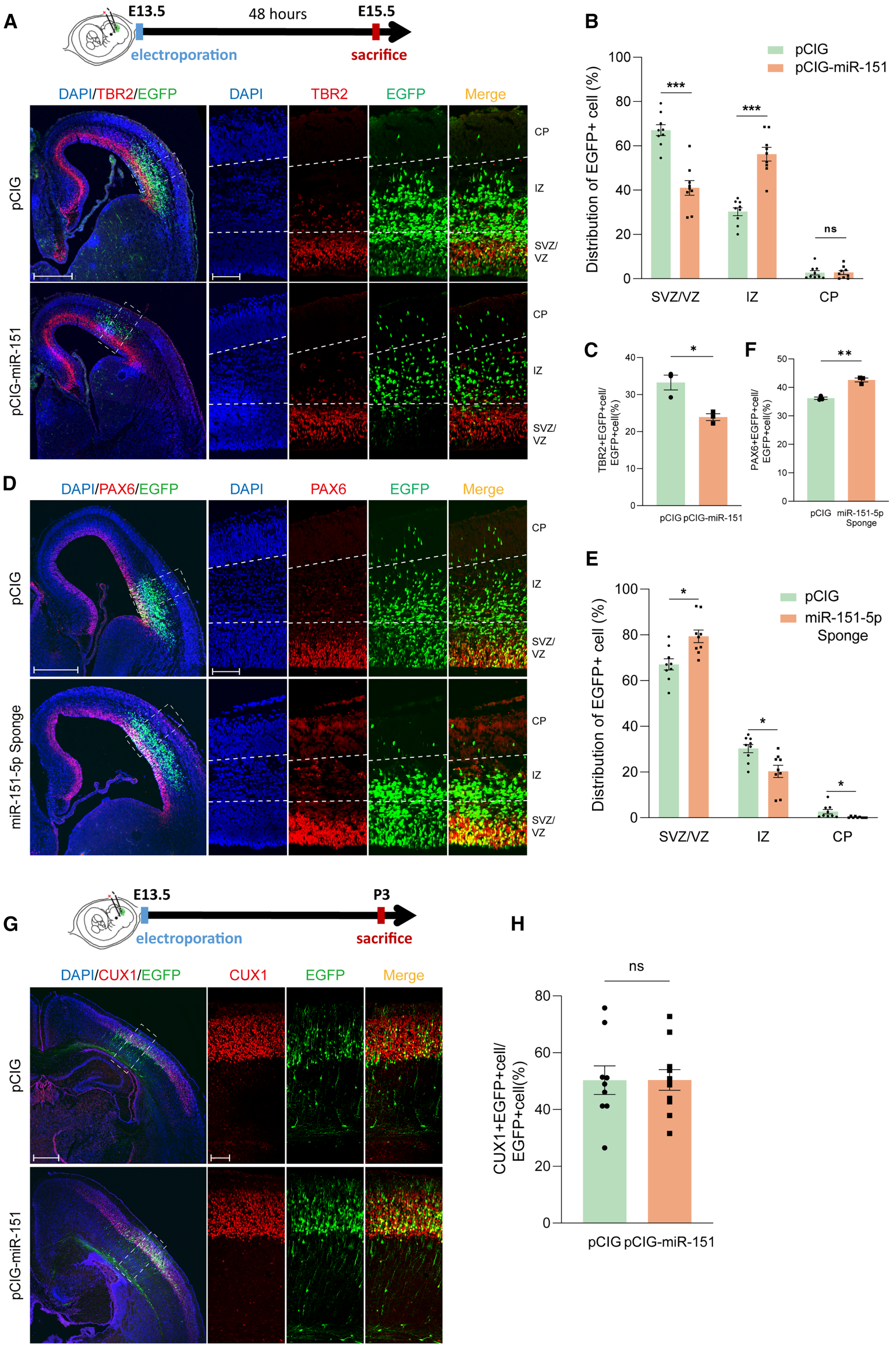

(legend on next page)

knockdown miR-151-5p. Knockdown resulted in significant accumulation of EGFP$^+$ cells in the VZ/SVZ (Figures 2D and 2E). Co-labeling analysis revealed that these retained cells maintained high levels of PAX6 and TBR2 expression, with a concurrent reduction in NEUROD2$^+$ neurons (Figures 2F and S1J–S1N). This indicates that the downregulation of miR-151-5p prevents progenitors from exiting the germinal zone, leading to a blockade in neuronal differentiation and the maintenance of the progenitor state.

To determine whether these migration effects alter final cortical lamination, we conducted long-term IUE (E13.5-P3). Neurons labeled at E13.5 typically populate cortical layers II–IV and express the layer marker CUX1. Quantification of CUX1$^+$ EGFP$^+$ cell proportions showed no significant differences in laminar distribution (Figures 2G and 2H). Thus, while miR-151-5p modulates NSC differentiation, it does not affect the ultimate laminar organization of the cerebral cortex.

Conditional knockout of miR-151-5p enhances NSC stemness and proliferative capacity

Based on prior findings, we generated miR-151 knockout mice by flanking the miR-151 locus with *loxP* sites within the intron spanning exons 22–23 of the *Ptk2* gene (Figure S2A). Crossing these mice with Emx1-IRES-Cre drivers (expressing Cre recombinase in dorsal telencephalon) yielded conditional knockouts (cko: fl/fl, cre+). RT-qPCR confirmed significantly reduced miR-151-5p expression in cko NSC versus control (ctrl, fl/fl; Cre-negative), validating successful knockout (Figure S2B). Meanwhile, we also assessed the expression of the host gene *Ptk2* to rule out potential *cis*-effects. RT-qPCR and western blot analyses demonstrated that miR-151 ablation did not alter *Ptk2* mRNA or protein levels (Figures S2C and S2D).

Morphometric analysis of P3 brains revealed no significant differences in cortical lamination or thickness between genotypes, as assessed by layer-specific markers: CUX1 (layers II–IV), CTIP2 (layer V), and TLE4 (layer VI) (Figures S2C and S2E). Furthermore, EdU pulse labeling at E14.5 followed by fate analysis at P3 showed unchanged proportions of CUX1$^+$EdU$^+$ neurons in cko mice (Figures S2F and S2G), indicating unaltered neuronal fate specification.

To investigate cell-autonomous functions, we isolated primary NSCs from E14.5 ctrl and cko embryos. cko NSCs exhibited significantly enhanced neurosphere formation (Figures 3A–3C) and elevated SOX2 protein expression (Figure 3D), indicating potentiated stemness. Combined EdU/immunofluorescence assays further demonstrated increased proliferation (SOX2$^+$EdU$^+$ cells) (Figures 3E, 3F, S3A, and S3B) and shifted differentiation potential, with reduced GFAP$^+$ astrocytes and increased MAP2$^+$ neurons in cko cultures (Figures 3G–3I). These findings were corroborated by RT-qPCR (Figures S3C and S3D), collectively indicating that miR-151-5p deletion enhances NSC proliferative capacity and alters differentiation trajectories.

miR-151-5p deletion accelerates NSC proliferation via S-phase shortening

While miR-151-5p knockout is known to alter NSC proliferation and differentiation *in vitro*, its *in vivo* functions during cortical development remained undefined. To address this, we assessed proliferative capacity in NSCs from E13.5 ctrl and cko dorsal cortex using 30 min EdU labeling combined with SOX2 immunofluorescence (Figure 4A). cko mice exhibited a significantly increased proportion of SOX2$^+$EdU$^+$ cells (Figure 4B), indicating enhanced NSC proliferation. Concurrently, TBR2 staining revealed altered differentiation dynamics post-knockout (Figures S4A and S4B). BrdU/EdU dual-pulse labeling was employed to dissect cell cycle changes (Figure 4C). Quantification of BrdU$^+$ EdU$^+$ (S-phase) and BrdU$^+$ EdU$^-$ (post-S-phase) populations using established cell cycle

Figure 2. miR-151-5p modulates NSC differentiation without altering cortical lamination

(A) Immunostaining of E15.5 brain sections electroporated with control (pCIG) or miR-151-OE plasmid on E13.5. White-dotted rectangles mark comparable regions for magnification.

(B) Quantitative analysis of the distribution of EGFP$^+$ cells after dividing the neocortex into three parts (SVZ/VZ, IZ, CP), n = 9 biological replicates.

(C) Quantification of the ratio of TBR2$^+$EGFP$^+$ cells in all EGFP$^+$ cells.

(D) Immunostaining of E15.5 brain sections electroporated with control (pCIG) or miR-151-5p Sponge plasmid on E13.5. White-dotted rectangles mark comparable regions for magnification.

(E) Quantitative analysis of the distribution of EGFP$^+$ cells after dividing the neocortex into three parts (SVZ/VZ, IZ, and CP) (n = 9 biological replicates).

(F) Quantification of the ratio of PAX6$^+$EGFP$^+$ cells in all EGFP$^+$ cells.

(G) Immunostaining of P3 brain sections electroporated with control or miR-151 plasmid on E13.5. White-dotted rectangles mark comparable regions for magnification.

(H) Quantitative analysis of the ratio of CUX1$^+$EGFP$^+$ cells in all CUX1$^+$ cells. Scale bars for the 10x images, 500 μm; 40x images, 100 μm. Data are presented as mean ± SEM. Individual data points represent independent biological replicates. Statistical analysis was performed by unpaired two-tailed Student's t test; ns, not significant; *$p < 0.05$; **$p < 0.01$; ***$p < 0.001$.

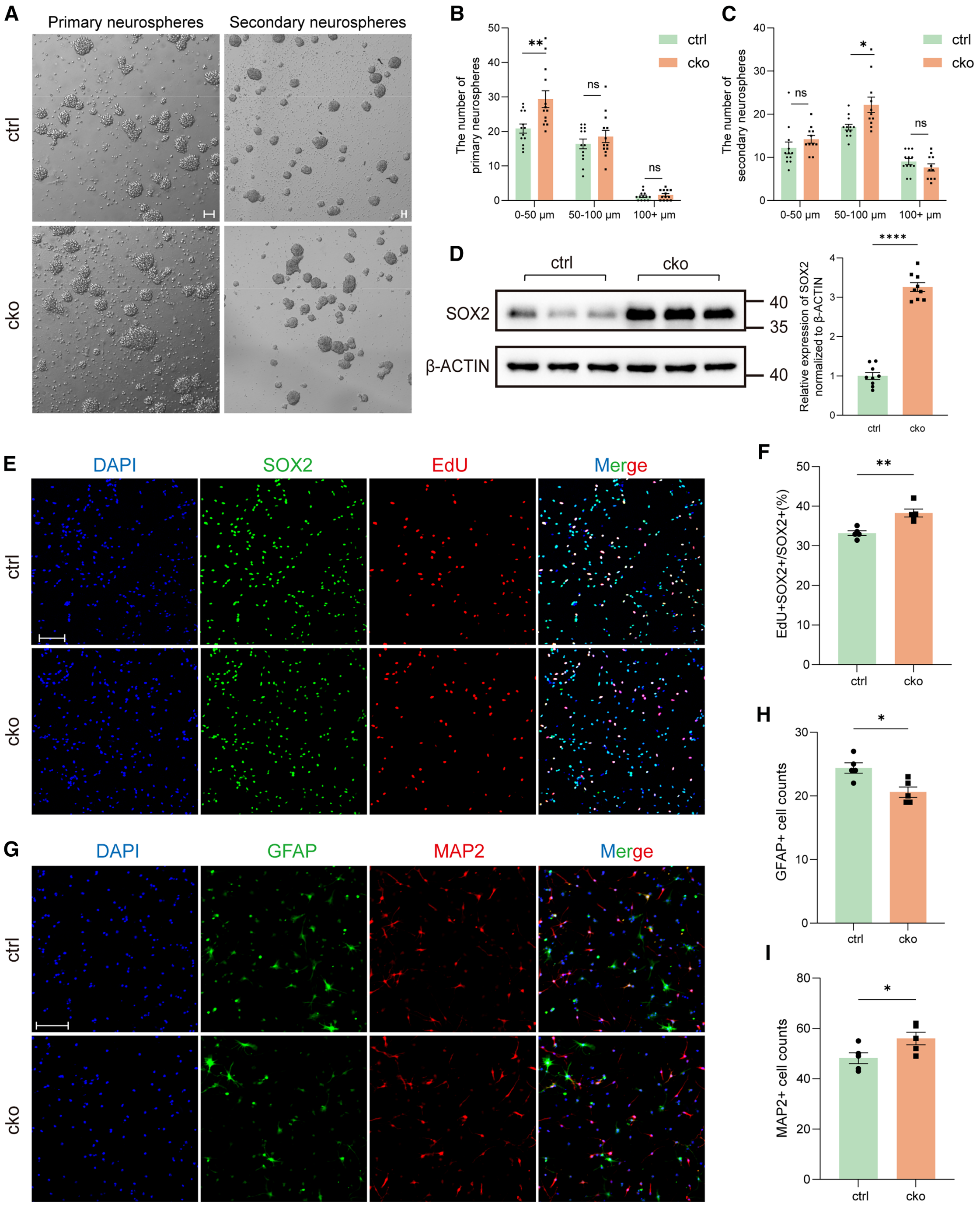

Figure 3. Conditional knockout of miR-151-5p enhances NSCs proliferative capacity and changes their differentiation potential
(A–C) Representative images and quantification of NSC neurospheres (primary and secondary passage) derived from E14.5 miR-151 cko and ctrl littermate mice. *X* axis: neurosphere radius (μm). *Y* axis: number of neurospheres. Scale bars, 50 μm.
(D) Western blot of stemness marker (SOX2) in NSCs from E14.5 miR-151 cko and ctrl littermate mice. β-ACTIN is used as a loading control. ImageJ is used to analyze the gray scale of signals.
(E) Detection of NSC stemness from E14.5 miR-151 cko and ctrl littermate mice by immunostaining.
(F) Quantitative analysis of the ratio of $EdU^{+}SOX2^{+}$ cells in all $SOX2^{+}$ cells, n = 5 biological replicates.

(legend continued on next page)

formulae demonstrated specific shortening of S-phase duration in cko NSCs, with unchanged G1/G2/M phases (Figures 4D and 4E). This suggests that accelerated proliferation stems from reduced DNA replication time.

Subsequently, we designed a "short-term" birthdating BrdU incorporation experimental protocol to assess neurogenesis during a short time window of 1 day. NSC that had entered the cell cycle within a 24-h period was labeled with EdU, while actively proliferating cells were identified by KI67 staining. The results of E13.5–14.5 EdU labeling (Figures 4F and 4G) and E14.5–15.5 EdU labeling (Figures S4C and S4D) all showed an increased proportion of $KI67^{+}EdU^{+}$ cells in the cko group, suggesting the NSC proliferative capacity was enhanced. Cortical development involves sequential neurogenic and gliogenic phases. To assess gliogenesis, we quantified $ALDH1L1^{+}$ astrocytes in cko mice at E18.5 and P3. Both time points revealed reduced astrocyte production (Figures S4E–S4H). Combined with prior *in vitro* data, these results demonstrate impaired astrocytic differentiation capacity following miR-151-5p deletion.

miR-151-5p regulates NSC proliferation via targeting APH1A/NICD axis

Building on our prior findings that miR-151-5p modulates NSC proliferation and differentiation, we investigated its regulatory mechanism. Multi-database miRNA target prediction consistently identified *Aph1a* as a top candidate (Figure 5A). To validate this interaction, we cloned the *Aph1a* 3′UTR downstream of the Renilla luciferase gene in the psiCHECK-2 vector. Transfection with miR-151-5p mimics resulted in a significant reduction in Renilla luciferase activity (Figure 5B), confirming that miR-151-5p directly targets the *Aph1a* 3′UTR to repress gene expression. Concordantly, western blot revealed elevated APH1A protein levels in E14.5 cko NSCs (Figure 5C), confirming miR-151-5p-mediated repression of APH1A. To functionally link APH1A to neural development, we overexpressed APH1A via E13.5 IUE. At E16.5, APH1A-overexpressing embryos exhibited increased cell retention in the VZ and reduced migration to the cortical plate (CP) (Figures 5D–5F), phenocopying miR-151-5p knockdown effects.

Given APH1A as a core subunit of γ-secretase—an aspartyl protease complex critical for Notch activation via NICD release (Yang et al., 2019; Zhou et al., 2019), we assessed pathway activity. Western blot showed elevated SOX2 and NICD protein levels in cko NSC (Figure 5G). Using a CBFRE-mcherry Notch activity reporter (Bultje et al., 2009; Mizutani et al., 2007), we observed intensified mcherry signals restricted to the VZ upon APH1A overexpression (Figures 5H–5J), indicating enhanced Notch activation. This demonstrates that APH1A amplifies Notch signaling to sustain NSC stemness and proliferation within the VZ.

Transcriptomic profiling reveals miR-151-5p orchestrates cell cycle and gliogenesis in NSCs

To elucidate genome-wide molecular mechanisms underlying neural developmental defects upon miR-151-5p deletion, we performed RNA-seq on NSCs isolated from miR-151 cko and ctrl littermates. Differential expression analysis (DESeq2; $|\log_2 FC| > 0.9$, p value < 0.01) identified 504 up-regulated and 915 down-regulated genes (Figure 6A). GO enrichment analysis (clusterProfiler) revealed that up-regulated genes were significantly enriched for cell division and cell cycle biological processes, while down-regulated genes clustered in gliogenesis and neurogenesis regulation (Figures 6B and 6C). Corresponding enrichments were observed in cellular component and molecular function terms (Figures S5A–S5D). KEGG pathway analysis further demonstrated up-regulated gene enrichment in cell cycle pathways, whereas down-regulated genes implicated cytoskeleton and cell adhesion pathways (Figures 6D and 6E; visualized in Figures S5E and S5F). These transcriptomic signatures robustly corroborated our functional data on NSC proliferation and differentiation defects.

miR-151-5p impacts adult mouse memory ability by targeting APH1A and is associated with Alzheimer's disease

As a core γ-secretase subunit, APH1A mediates both NOTCH cleavage and amyloid-beta (Aβ) processing—a key pathway in Alzheimer's disease (AD) pathogenesis. To determine whether the functional abnormalities observed in NSCs during embryonic development translate into long-term functional deficits in adulthood, we performed contextual fear conditioning on 8-week-old male mice. Emx1-miR-151 cko mice exhibited significantly reduced freezing duration versus controls (Figure 7A), indicating impaired fear memory—a deficit consistent with AD phenotypes (Forner et al., 2021; Jahn, 2013). Post-behavioral validation confirmed sustained miR-151-5p downregulation (Figure 7B) and APH1A elevation (Figure 7C) in cko brains. Furthermore, we analyzed *Aph1a* expression in the prefrontal cortex of normal individuals and AD patients

(G) Detection of NSC differentiation potential from E16.5 miR-151 cko and ctrl littermate mice by immunostaining.
(H and I) Quantitative analysis of the number of $GFAP^{+}$ and $MAP2^{+}$ cells, $n = 5$ biological replicates. Scale bars, 100 μm. Data are presented as mean ± SEM. Individual data points represent independent biological replicates. Statistical analysis was performed by unpaired two-tailed Student's t test; ns, not significant; *$p < 0.05$; **$p < 0.01$; ****$p < 0.0001$.

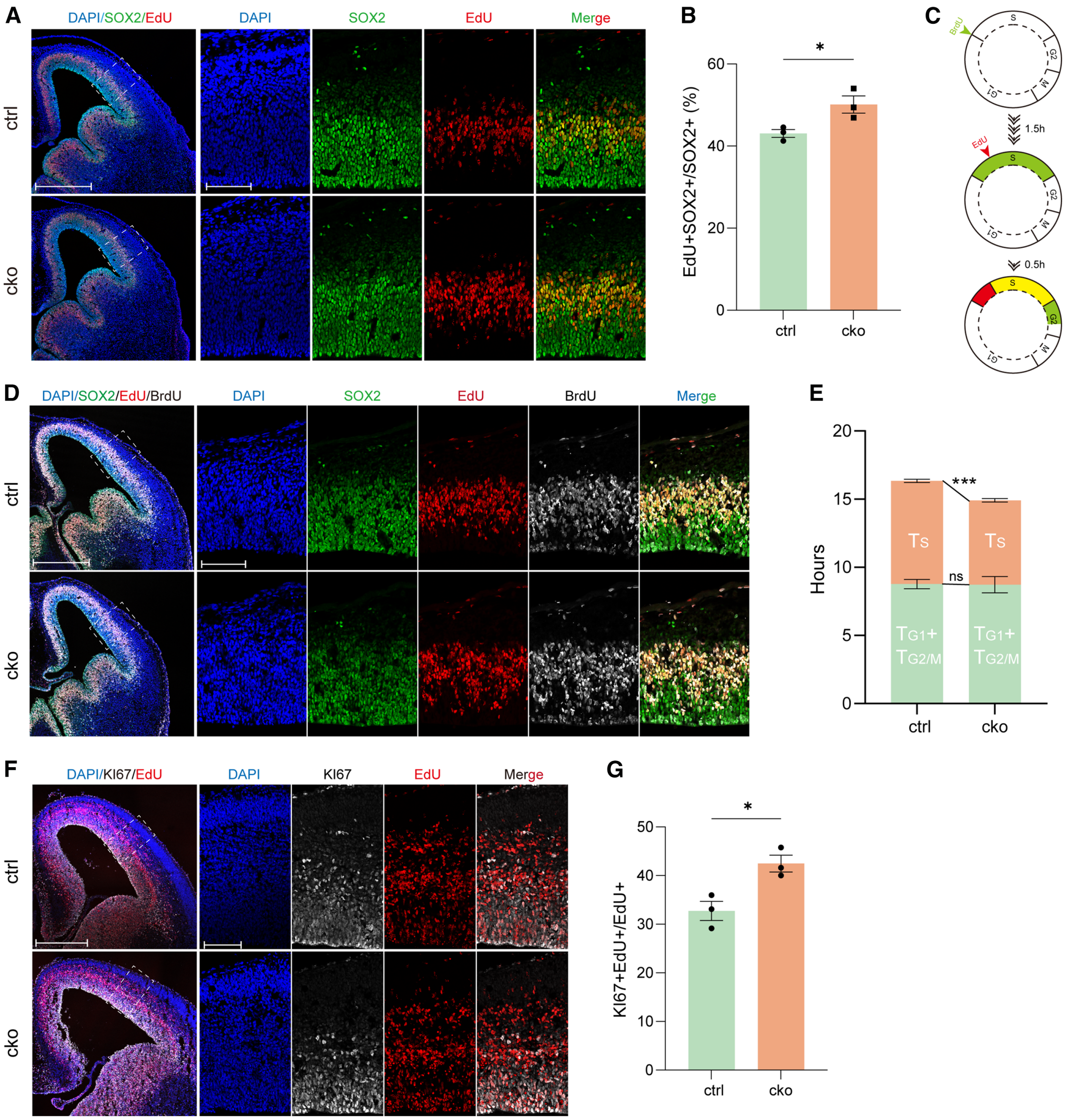

Figure 4. miR-151-5p deletion accelerates NSC proliferation via S-phase shortening

(A) Immunostaining of E13.5 brain sections derived from miR-151 cko and ctrl littermate mice. White-dotted rectangles mark comparable regions for magnification.

(B) Quantitative analysis of the ratio of $EdU^{+}SOX2^{+}$ cells in all $SOX2^{+}$ cells.

(C) Scheme of the experimental analysis of the cell cycle.

(D) Immunostaining of E13.5 coronal sections derived from miR-151 cko and ctrl littermate mice. White-dotted rectangles mark comparable regions for magnification.

(E) Quantification of NSCs in different phases of the cell cycle in the neocortex of ctrl and cko mice.

(F) Immunostaining of E14.5 coronal sections derived from miR-151 cko and ctrl littermate mice. White-dotted rectangles mark comparable regions for magnification.

(G) Quantification of the ratio of $KI67^{+}EdU^{+}$ cells in all EdU^{+} cells. Scale bars for the 10x images, 500 μm; 40x images, 100 μm. Data are presented as mean ± SEM. Individual data points represent independent biological replicates. Statistical analysis was performed by unpaired two-tailed Student's *t* test, $n = 3$ biological replicates; ns, not significant; $*p < 0.05$; $***p < 0.001$.

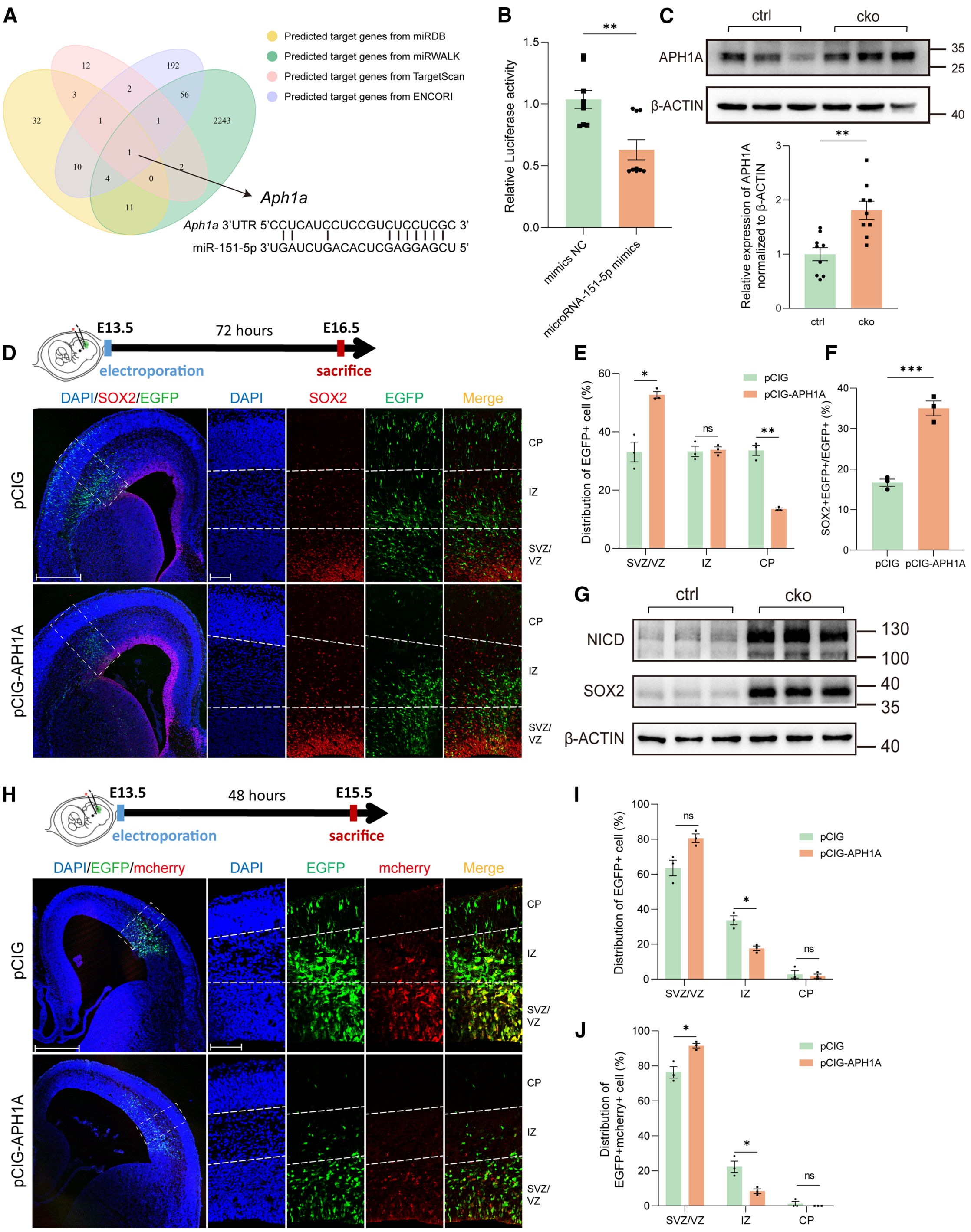

Figure 5. miR-151-5p regulates NSC proliferation via targeting APH1A/NICD axis

(A) Screening for downstream targets of miR-151-5p by four miRNA database and the intersection analysis identified the *Aph1a*.

(B) Measurements of luciferase activity after transfection with miR-151 mimics and control.

(C) Western blot of APH1A in NSCs from E14.5 miR-151 cko and ctrl littermate mice. β-ACTIN is used as a loading control. ImageJ is used to analyze the gray scale of signals.

(D) Immunostaining of E16.5 brain sections electroporated with control or APH1A-OE plasmid on E13.5. White-dotted rectangles mark comparable regions for magnification.

(legend continued on next page)

using the AlzData. In GSE5281, we found that *Aph1a* expression was significantly higher in the prefrontal cortex of AD patients compared to NC (Figure 7D), suggesting that *Aph1a* may be involved in the pathogenesis of AD.

In summary, this study demonstrates that miR-151-5p modulates γ-secretase function by targeting APH1A through overexpression and knockout experiments. This regulation establishes a spatial Notch signaling gradient within the VZ, thereby modulating the proliferation and differentiation potential of NSCs (Figure 7E).

DISCUSSION

In this study, we identify a critical role for the evolutionarily conserved miRNA, miR-151-5p, in mouse cortical neurogenesis. Our findings demonstrate that miR-151-5p is expressed in NSCs, and it functions as a molecular brake on proliferation and promotes neuronal differentiation by negatively regulating its direct target, APH1A, a key regulator of the Notch signaling pathway.

Evolutionarily, miR-151-5p appears to be a mammalian-specific innovation. Our data indicate that while the flanking intronic sequences of the *Ptk2* gene are highly divergent, the miR-151-5p sequence itself remains perfectly conserved across mammalian species, underscoring its functional importance. Given that the host gene *Ptk2* is known to play critical roles in cortical development and neuronal morphology (Beggs et al., 2003; Rico et al., 2004), it was imperative to rule out any off-target effects on its expression. We confirmed that the conditional deletion of miR-151 did not alter the transcriptional or translational levels of *Ptk2*. This validation ensures that the observed phenotypes are specific to miR-151-5p deficiency and are not driven by the disruption of the host gene.

While the expression and function of miRNAs in different neocortical cell types have been extensively studied, miR-151-5p remained poorly studied. Recent advances demonstrate that miRNAs exhibit different expression and functional specificity across various neocortical cell types. For instance, miRNAs such as miR-124 and miR-132 are specifically expressed in neurons, primarily governing synaptic plasticity and identity maintenance (Eugene et al., 2007; Walgrave et al., 2021). Others, including miR-155, miR-219, and miR-146a, exhibit glial-specific expression—regulating inflammation, myelination, and immune responses in astrocytes, oligodendrocytes, and microglia, respectively (Dugas et al., 2010; Fan et al., 2017; Pena-Philippides et al., 2016; Su et al., 2017). Similarly, miRNAs like miR-9 and miR-210 are expressed in NSCs and play important roles in maintaining NSC stemness and regulating differentiation (Coolen et al., 2012, 2013; Fasanaro et al., 2008). Furthermore, research into miR-151-3p has demonstrated significant promise as a neuroimaging diagnostic biomarker for major depressive disorder (MDD), potentially offering a novel tool for the standardization of clinical diagnosis in MDD (Liang et al., 2025). Our findings establish that miR-151-5p is enriched in NSC and constrains their proliferation while promoting neuronal differentiation during neural development.

RNA-seq analysis revealed significant enrichment of cell cycle and DNA replication-related KEGG pathways among genes upregulated upon miR-151-5p knockout. Given the established neurodevelopmental expression pattern and functional role of miR-151-5p, future studies will prioritize identifying its target genes governing mitotic progression and nuclear positioning to delineate molecular mechanisms regulating cell division.

During screening for miR-151-5p target genes, we identified several candidates closely associated with the Notch signaling pathway and neurogenesis (e.g., *N4bp1* and *Aph1a*). Specifically, by suppressing APH1A, miR-151-5p modulates γ-secretase activity and consequently reduces the production of NICD. This provides a post-transcriptional mechanism for fine-tuning the spatiotemporal Notch signaling gradient within the VZ, ultimately controlling the balance between NSC self-renewal and differentiation. Intriguingly, the impaired fear memory observed in adult miR-151-5p cko mice aligns with a previous report linking the miR-151-5p/APH1A axis to fear memory formation in the hippocampus (Xu et al., 2019). This functional conservation suggests that the miR-151-5p/APH1A/Notch regulatory axis exerts a lasting

(E) Quantitative analysis of the distribution of EGFP$^+$ cells after dividing the neocortex into three parts (SVZ/VZ, IZ, and CP).
(F) Quantification of the ratio of SOXx2$^+$EGFP$^+$ cells in all EGFP$^+$ cells.
(G) Western blot of neural stem marker (SOX2 and NICD) in NSCs from E14.5 miR-151 cko and ctrl littermate mice. β-ACTIN is used as a loading control.
(H) Immunostaining of E15.5 brain sections electroporated with control or APH1A-OE and CBFRE-mcherry plasmid on E13.5. White-dotted rectangles mark comparable regions for magnification.
(I and J) Quantitative analysis of the distribution of EGFP$^+$ cells (I) and mcherry$^+$ EGFP$^+$ cells (J) after dividing the neocortex into three parts (SVZ/VZ, IZ, and CP). Scale bars for the 10x images, 500 μm; 40x images, 100 μm. Data are presented as mean ± SEM. Individual data points represent independent biological replicates. Statistical analysis was performed by unpaired two-tailed Student's t test, $n = 3$ biological replicates; ns, not significant; *$p < 0.05$; **$p < 0.01$; ***$p < 0.001$.

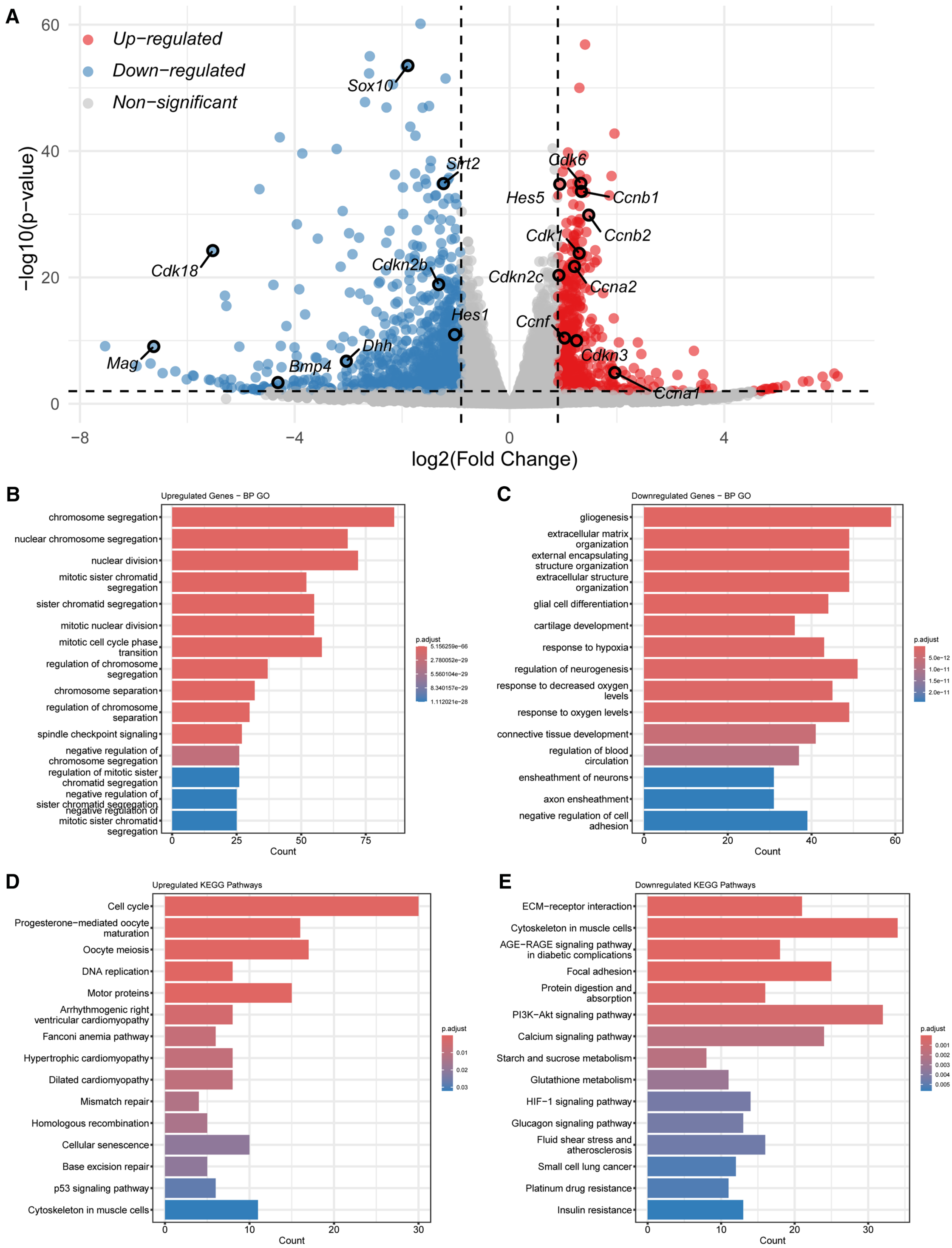

Figure 6. Transcriptomic profiling reveals miR-151-5p orchestrates cell cycle and gliogenesis in NSCs

(A) Volcano plot of genes upregulated (red) and downregulated (blue) in E14.5 miR-151 cko NSCs compared with control, as analyzed using DESeq2.

(B) The GO enrichment results of biological processes were obtained by using up-regulated genes, as analyzed using clusterProfiler.

(C) The GO enrichment results of biological processes were obtained by using down-regulated genes, as analyzed using clusterProfiler.

(legend continued on next page)

impact beyond embryonic development, potentially contributing to cognitive functions and implicating it in the pathophysiology of neurodevelopmental and neurodegenerative disorders such as AD.

In conclusion, our study elucidates a novel regulatory pathway in which miR-151-5p, by targeting APH1A, modulates Notch signaling to govern NSC fate determination during cortical development. Collectively, these findings not only deepen our understanding of the molecular orchestration of neurogenesis but also establish a functional link between neurodevelopmental regulatory axis and adult cognitive function and associated disorders.

RESOURCE AVAILABILITY

Lead contact

Further information and requests for resources and reagents should be directed to and will be fulfilled by the lead contact, Xiaozhong Peng (pengxiaozhong@pumc.edu.cn).

Materials availability

The mouse strains and related materials generated in this study are available upon request from the lead contact.

Data and code availability

The RNA-seq data generated in this study have been deposited in GEO (GSE327779). No original code is involved. Additional information required to reanalyze the data in this study is available from the lead contact upon request.

ACKNOWLEDGMENTS

This work was supported by the National Key Research and Development Program of China (2022YFA1103803), the CAMS Innovation Fund for Medical Sciences (CIFMS; 2021-I2M-1-024), the CAMS Basic Research Fund (2024-RW310-01), State Key Laboratory Special Fund 2060204, and Overseas Expertise Introduction Center for Discipline Innovation (“111Center”) (BP0820029). We thank State Key Laboratory of Common Mechanism Research of Major Diseases Platform for consultation and instrument availability that supported this work.

AUTHOR CONTRIBUTIONS

All authors participated in the scientific discussion. X.P. and W.L. conceived the research; X.P., W.L., P.S., X.W., and L.L. designed the experiments; X.W., L.L., Z.C., Y.Z., B.Y., and L.H. carried out the experimental studies and analyses; X.W. performed bioinformatics analysis; X.W. and W.L. wrote the manuscript; X.P. and W.L. revised the manuscript. All authors commented on the manuscript.

DECLARATION OF INTERESTS

The authors declare no competing interests.

STAR★METHODS

Detailed methods are provided in the online version of this paper and include the following:

- KEY RESOURCES TABLE
- EXPERIMENTAL MODEL AND STUDY PARTICIPANT DETAILS
  - Experimental animals
  - Bacterial strains and culture conditions
  - Cell culture
- METHOD DETAILS
  - Tissue section
  - Immunofluorescence
  - 5-Ethynyl-2′-deoxyuridine (EdU) staining
  - In utero electroporation
  - Neurosphere formation assay for NSCs
  - Western blot
  - RT−qPCR
  - Luciferase assays
  - Fear conditioning behavioral experiment
  - RNA seq
- QUANTIFICATION AND STATISTICAL ANALYSIS
  - Image analysis, quantification and statistical analysis

SUPPLEMENTAL INFORMATION

Supplemental information can be found online at https://doi.org/10.1016/j.stemcr.2026.102927.

Received: September 4, 2025
Revised: April 19, 2026
Accepted: April 19, 2026
Published: May 21, 2026

REFERENCES

Bartel, D.P. (2009). MicroRNAs: target recognition and regulatory functions. Cell *136*, 215–233. https://doi.org/10.1016/j.cell.2009.01.002.

Beggs, H.E., Schahin-Reed, D., Zang, K., Goebbels, S., Nave, K.A., Gorski, J., Jones, K.R., Sretavan, D., and Reichardt, L.F. (2003). FAK deficiency in cells contributing to the basal lamina results in cortical abnormalities resembling congenital muscular dystrophies. Neuron *40*, 501–514. https://doi.org/10.1016/s0896-6273(03)00666-4.

Bultje, R.S., Castaneda-Castellanos, D.R., Jan, L.Y., Jan, Y.-N., Kriegstein, A.R., and Shi, S.-H. (2009). Mammalian Par3 regulates

(D) The KEGG-pathway enrichment results of biological processes were obtained by using up-regulated genes, as analyzed using clusterProfiler.

(E) The KEGG-pathway enrichment results of biological processes were obtained by using down-regulated genes, as analyzed using clusterProfiler.

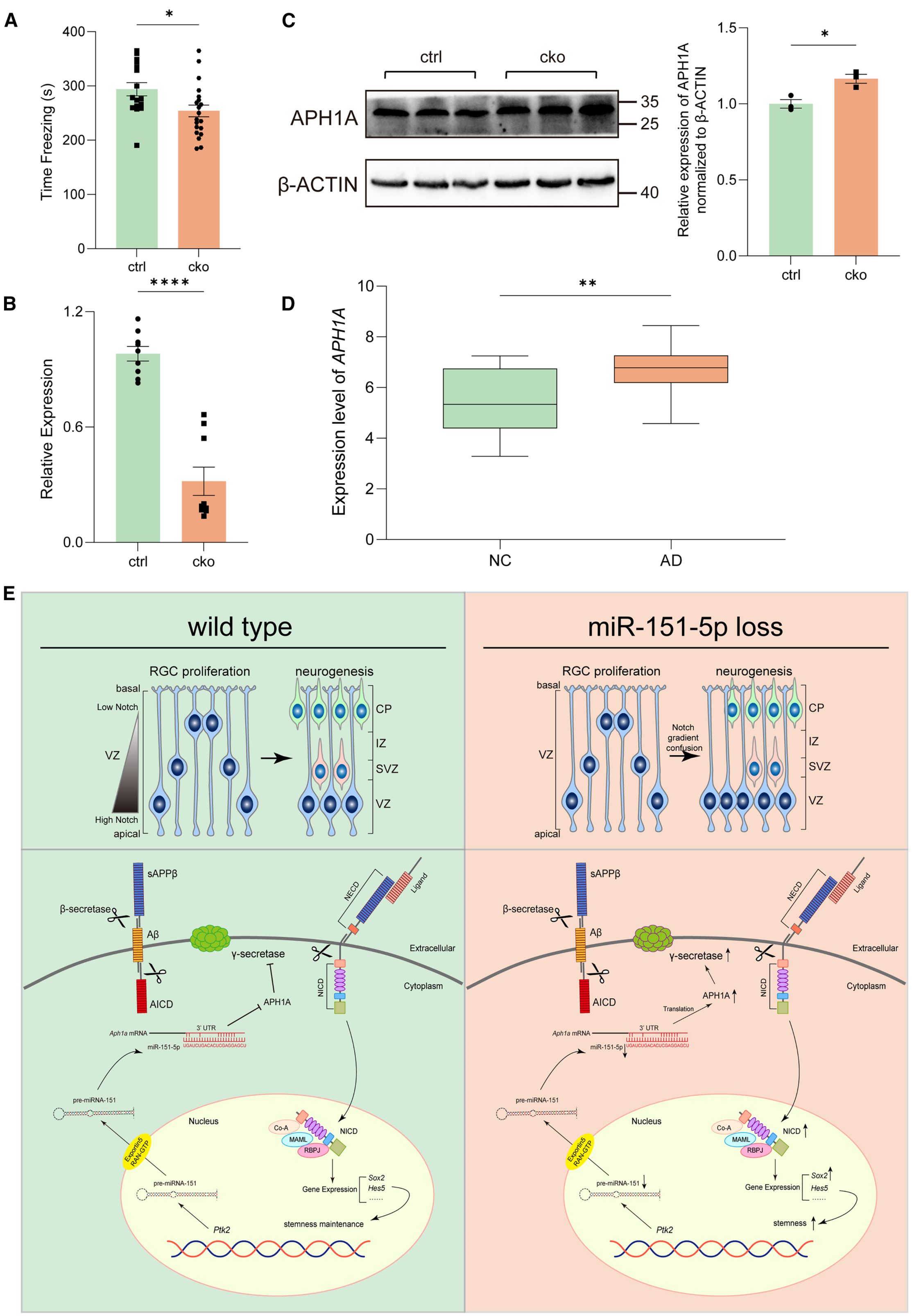

(legend on next page)

progenitor cell asymmetric division via notch signaling in the developing neocortex. Neuron *63*, 189–202. https://doi.org/10.1016/j.neuron.2009.07.004.

Chen, C., Wang, D., Moshaverinia, A., Liu, D., Kou, X., Yu, W., Yang, R., Sun, L., and Shi, S. (2017). Mesenchymal stem cell transplantation in tight-skin mice identifies miR-151-5p as a therapeutic target for systemic sclerosis. Cell Res. *27*, 559–577. https://doi.org/10.1038/cr.2017.11.

Coolen, M., Katz, S., and Bally-Cuif, L. (2013). miR-9: a versatile regulator of neurogenesis. Front. Cell. Neurosci. *7*, 220. https://doi.org/10.3389/fncel.2013.00220.

Coolen, M., Thieffry, D., Drivenes, Ø., Becker, T.S., and Bally-Cuif, L. (2012). miR-9 controls the timing of neurogenesis through the direct inhibition of antagonistic factors. Dev. Cell *22*, 1052–1064. https://doi.org/10.1016/j.devcel.2012.03.003.

Del Bene, F., Wehman, A.M., Link, B.A., and Baier, H. (2008). Regulation of neurogenesis by interkinetic nuclear migration through an apical-basal notch gradient. Cell *134*, 1055–1065. https://doi.org/10.1016/j.cell.2008.07.017.

Di Bella, D.J., Habibi, E., Stickels, R.R., Scalia, G., Brown, J., Yadollahpour, P., Yang, S.M., Abbate, C., Biancalani, T., Macosko, E.Z., et al. (2021). Molecular logic of cellular diversification in the mouse cerebral cortex. Nature *595*, 554–559. https://doi.org/10.1038/s41586-021-03670-5.

Ding, J., Huang, S., Wu, S., Zhao, Y., Liang, L., Yan, M., Ge, C., Yao, J., Chen, T., Wan, D., et al. (2010). Gain of miR-151 on chromosome 8q24.3 facilitates tumour cell migration and spreading through downregulating RhoGDIA. Nat. Cell Biol. *12*, 390–399. https://doi.org/10.1038/ncb2039.

Dugas, J.C., Cuellar, T.L., Scholze, A., Ason, B., Ibrahim, A., Emery, B., Zamanian, J.L., Foo, L.C., McManus, M.T., and Barres, B.A. (2010). Dicer1 and miR-219 are required for normal oligodendrocyte differentiation and myelination. Neuron *65*, 597–611. https://doi.org/10.1016/j.neuron.2010.01.027.

Eugene, V.M., Jiangwen, Z., Monica, A.C., and Tom, M. (2007). The MicroRNA miR-124 promotes neuronal differentiation by triggering brain-specific alternative pre-mRNA splicing. Mol. Cell *27*, 435–448. https://doi.org/10.1016/j.molcel.2007.07.015.

Fan, H.-B., Chen, L.-X., Qu, X.-B., Ren, C.-L., Wu, X.-X., Dong, F.-X., Zhang, B.-L., Gao, D.-S., and Yao, R.-Q. (2017). Transplanted miR-219-overexpressing oligodendrocyte precursor cells promoted remyelination and improved functional recovery in a chronic demyelinated model. Sci. Rep. *7*, 41407. https://doi.org/10.1038/srep41407.

Fasanaro, P., D'Alessandra, Y., Di Stefano, V., Melchionna, R., Romani, S., Pompilio, G., Capogrossi, M.C., and Martelli, F. (2008). MicroRNA-210 modulates endothelial cell response to hypoxia and inhibits the receptor tyrosine kinase ligand ephrin-A3. J. Biol. Chem. *283*, 15878–15883. https://doi.org/10.1074/jbc.M800731200.

Fenoglio, C., Ridolfi, E., Galimberti, D., and Scarpini, E. (2013). An emerging role for long non-coding RNA dysregulation in neurological disorders. Int. J. Mol. Sci. *14*, 20427–20442. https://doi.org/10.3390/ijms141020427.

Fiddes, I.T., Lodewijk, G.A., Mooring, M., Bosworth, C.M., Ewing, A.D., Mantalas, G.L., Novak, A.M., van den Bout, A., Bishara, A., Rosenkrantz, J.L., et al. (2018). Human-specific NOTCH2NL genes affect notch signaling and cortical neurogenesis. Cell *173*, 1356–1369.e22. https://doi.org/10.1016/j.cell.2018.03.051.

Forner, S., Kawauchi, S., Balderrama-Gutierrez, G., Kramár, E.A., Matheos, D.P., Phan, J., Javonillo, D.I., Tran, K.M., Hingco, E., da Cunha, C., et al. (2021). Systematic phenotyping and characterization of the 5xFAD mouse model of Alzheimer's disease. Sci. Data *8*, 270. https://doi.org/10.1038/s41597-021-01054-y.

Gal, J.S., Morozov, Y.M., Ayoub, A.E., Chatterjee, M., Rakic, P., and Haydar, T.F. (2006). Molecular and morphological heterogeneity of neural precursors in the mouse neocortical proliferative zones. J. Neurosci. *26*, 1045–1056. https://doi.org/10.1523/JNEUROSCI.4499-05.2006.

Götz, M., and Huttner, W.B. (2005). The cell biology of neurogenesis. Nat. Rev. Mol. Cell Biol. *6*, 777–788. https://doi.org/10.1038/nrm1739.

Hippenmeyer, S. (2023). Principles of neural stem cell lineage progression: insights from developing cerebral cortex. Curr. Opin. Neurobiol. *79*, 102695. https://doi.org/10.1016/j.conb.2023.102695.

Huang, F., Ren, Y., Hua, Y., Wang, Y., Li, R., Ji, N., Zeng, X., Bai, D., Chen, Q., Zhou, X., et al. (2024). m6A-dependent mature miR-151-5p accelerates the malignant process of HNSCC by targeting

Figure 7. miR-151-5p impacts adult mouse memory ability by targeting APH1A and is associated with AD

(A) Quantitative analysis of the freezing time in the fear conditioning experiment ($n = 16$ for the ctrl group, $n = 20$ for the miR-151 cko group).

(B) RT-qPCR measurement of miR-151-5p expression level with ctrl and cko mice dorsal cortex after FC experiment, $n = 3$ biological replicates.

(C) Western blot of APH1A in dorsal cortex from ctrl and cko mice after FC experiment, β-actin is used as a loading control. ImageJ is used to analyze the gray scale of signals.

(D) The expression of *Aph1a* in the frontal cortex of normal individuals and AD patients is analyzed by using the NCBI dataset GSE5281.

(E) A cartoon showing how miR-151-5p regulates NICD to control radial glial cell proliferation or differentiation in the developing mammalian neocortex. miR-151-5p affects the function of γ-secretase by targeting APH1A, which in turn affects the production of NICD, which in turn affects the stemness maintenance and proliferation ability of NSCs. At the same time, miR-151-5p may affect the cell cycle of NSC by affecting the Notch concentration gradient in VZ, and regulate the stemness maintenance and proliferation ability. Data are presented as mean ± SEM. Individual data points represent independent biological replicates. Statistical analysis was performed by unpaired two-tailed Student's t test; $^{*}p < 0.05$; $^{**}p < 0.01$; $^{****}p < 0.0001$.

LYPD3. Mol. Biomed. *5*, 27. https://doi.org/10.1186/s43556-024-00189-9.

Jahn, H. (2013). Memory loss in Alzheimer's disease. Dialogues Clin. Neurosci. *15*, 445–454. https://doi.org/10.31887/DCNS.2013.15.4/hjahn.

Jorstad, N.L., Close, J., Johansen, N., Yanny, A.M., Barkan, E.R., Travaglini, K.J., Bertagnolli, D., Campos, J., Casper, T., Crichton, K., et al. (2023). Transcriptomic cytoarchitecture reveals principles of human neocortex organization. Science *382*, eadf6812. https://doi.org/10.1126/science.adf6812.

Kalogeropoulou, A., Lygerou, Z., and Taraviras, S. (2019). Cortical development and brain malformations: insights from the differential regulation of early events of DNA replication. Front. Cell Dev. Biol. *7*, 29. https://doi.org/10.3389/fcell.2019.00029.

Kirwan, P., Turner-Bridger, B., Peter, M., Momoh, A., Arambepola, D., Robinson, H.P.C., and Livesey, F.J. (2015). Development and function of human cerebral cortex neural networks from pluripotent stem cells in vitro. Dev. (camb. Engl.) *142*, 3178–3187. https://doi.org/10.1242/dev.123851.

Lewis, E.M.A., Kaushik, K., Sandoval, L.A., Antony, I., Dietmann, S., and Kroll, K.L. (2021). Epigenetic regulation during human cortical development: seq-ing answers from the brain to the organoid. Neurochem. Int. *147*, 105039. https://doi.org/10.1016/j.neuint.2021.105039.

Liang, W., Hou, L., Wang, W., Wang, B., Sun, C., Zhang, Y., Li, Z., Shi, R., Zhou, W., Tang, Y., et al. (2025). Alterations in miR-151a-3p of plasma-derived exosomes and associated multimodal neuroimaging patterns in major depressive disorder. Mol. Psychiatry *30*, 5177–5191. https://doi.org/10.1038/s41380-025-03102-0.

Liu, S., Dou, L., Miao, M., Man, X., Wei, B., Jiang, Z., Ouyang, Y., Ozaki, T., Yu, M., and Zhu, Y. (2023). HES1-mediated down-regulation of miR-138 sustains NOTCH1 activation and promotes proliferation and invasion in renal cell carcinoma. J. Exp. Clin. Cancer Res. *42*, 72. https://doi.org/10.1186/s13046-023-02625-0.

Ma, Q., Zhang, L., and Pearce, W.J. (2019). MicroRNAs in brain development and cerebrovascular pathophysiology. Am. J. Physiol. Cell Physiol. *317*, C3–C19. https://doi.org/10.1152/ajpcell.00022.2019.

Ma, Z., Zeng, Y., Wang, M., Liu, W., Zhou, J., Wu, C., Hou, L., Yin, B., Qiang, B., Shu, P., and Peng, X. (2023). N4BP1 mediates RAM domain-dependent notch signaling turnover during neocortical development. EMBO J. *42*, e113383. https://doi.org/10.15252/embj.2022113383.

Masui, S., Nakatake, Y., Toyooka, Y., Shimosato, D., Yagi, R., Takahashi, K., Okochi, H., Okuda, A., Matoba, R., Sharov, A.A., et al. (2007). Pluripotency governed by Sox2 via regulation of Oct3/4 expression in mouse embryonic stem cells. Nat. Cell Biol. *9*, 625–635. https://doi.org/10.1038/ncb1589.

McLaren, M., and Butts, J. (2025). Notch signaling in neurogenesis. Development *152*, dev204589. https://doi.org/10.1242/dev.204589.

Megason, S.G., and McMahon, A.P. (2002). A mitogen gradient of dorsal midline Wnts organizes growth in the CNS. Development *129*, 2087–2098. https://doi.org/10.1242/dev.129.9.2087.

Mizutani, K.i., Yoon, K., Dang, L., Tokunaga, A., and Gaiano, N. (2007). Differential Notch signalling distinguishes neural stem cells from intermediate progenitors. NATURE *449*, 351–355. https://doi.org/10.1038/nature06090.

Mohamed Ariff, I., Mitra, A., and Basu, A. (2012). Epigenetic regulation of self-renewal and fate determination in neural stem cells. J. Neurosci. Res. *90*, 529–539. https://doi.org/10.1002/jnr.22804.

Pena-Philippides, J.C., Caballero-Garrido, E., Lordkipanidze, T., and Roitbak, T. (2016). In vivo inhibition of miR-155 significantly alters post-stroke inflammatory response. J. Neuroinflammation *13*, 287. https://doi.org/10.1186/s12974-016-0753-x.

Rico, B., Beggs, H.E., Schahin-Reed, D., Kimes, N., Schmidt, A., and Reichardt, L.F. (2004). Control of axonal branching and synapse formation by focal adhesion kinase. Nat. Neurosci. *7*, 1059–1069. https://doi.org/10.1038/nn1317.

Rosebrock, D., Arora, S., Mutukula, N., Volkman, R., Gralinska, E., Balaskas, A., Aragonés Hernández, A., Buschow, R., Brändl, B., Müller, F.-J., et al. (2022). Enhanced cortical neural stem cell identity through short SMAD and WNT inhibition in human cerebral organoids facilitates emergence of outer radial glial cells. Nat. Cell Biol. *24*, 981–995. https://doi.org/10.1038/s41556-022-00929-5.

Sansom, S.N., Griffiths, D.S., Faedo, A., Kleinjan, D.J., Ruan, Y., Smith, J., van Heyningen, V., Rubenstein, J.L., and Livesey, F.J. (2009). The level of the transcription factor Pax6 is essential for controlling the balance between neural stem cell self-renewal and neurogenesis. PLoS Genet. *5*, e1000511. https://doi.org/10.1371/journal.pgen.1000511.

Shimojo, H., Ohtsuka, T., and Kageyama, R. (2008). Oscillations in notch signaling regulate maintenance of neural progenitors. Neuron *58*, 52–64. https://doi.org/10.1016/j.neuron.2008.02.014.

Shu, P., Wu, C., Liu, W., Ruan, X., Liu, C., Hou, L., Zeng, Y., Fu, H., Wang, M., Chen, P., et al. (2019a). The spatiotemporal expression pattern of microRNAs in the developing mouse nervous system. J. Biol. Chem. *294*, 3444–3453. https://doi.org/10.1074/jbc.RA118.004390.

Shu, P., Wu, C., Ruan, X., Liu, W., Hou, L., Fu, H., Wang, M., Liu, C., Zeng, Y., Chen, P., et al. (2019b). Opposing Gradients of MicroRNA Expression Temporally Pattern Layer Formation in the Developing Neocortex. Dev. Cell *49*, 764–785.e4. https://doi.org/10.1016/j.devcel.2019.04.017.

Su, Z.F., Sun, Z.W., Zhang, Y., Wang, S., Yu, Q.G., and Wu, Z.B. (2017). Regulatory effects of miR-146a/b on the function of endothelial progenitor cells in acute ischemic stroke in mice. Kaohsiung J. Med. Sci. *33*, 369–378. https://doi.org/10.1016/j.kjms.2017.05.010.

Sun, E., and Shi, Y. (2015). MicroRNAs: small molecules with big roles in neurodevelopment and diseases. Exp. Neurol. *268*, 46–53. https://doi.org/10.1016/j.expneurol.2014.08.005.

Sun, L., Zhao, L., Yang, G., Yan, C., Zhou, R., Zhou, X., Xie, T., Zhao, Y., Wu, S., Li, X., and Shi, Y. (2015). Structural basis of human γ-secretase assembly. Proc. Natl. Acad. Sci. USA *112*, 6003–6008. https://doi.org/10.1073/pnas.1506242112.

Telley, L., Agirman, G., Prados, J., Amberg, N., Fièvre, S., Oberst, P., Bartolini, G., Vitali, I., Cadilhac, C., Hippenmeyer, S., et al. (2019).

Temporal patterning of apical progenitors and their daughter neurons in the developing neocortex. Science *364*, eaav2522. https://doi.org/10.1126/science.aav2522.

Tolia, A., and De Strooper, B. (2009). Structure and function of γ-secretase. Semin. Cell Dev. Biol. *20*, 211–218. https://doi.org/10.1016/j.semcdb.2008.10.007.

Walgrave, H., Balusu, S., Snoeck, S., Vanden Eynden, E., Craessaerts, K., Thrupp, N., Wolfs, L., Horré, K., Fourne, Y., Ronisz, A., et al. (2021). Restoring miR-132 expression rescues adult hippocampal neurogenesis and memory deficits in alzheimer's disease. Cell Stem Cell *28*, 1805–1821.e8. https://doi.org/10.1016/j.stem.2021.05.001.

Wang, L., Wang, C., Moriano, J.A., Chen, S., Zuo, G., Cebrián-Silla, A., Zhang, S., Mukhtar, T., Wang, S., Song, M., et al. (2025). Molecular and cellular dynamics of the developing human neocortex. Nature *647*, 169–178. https://doi.org/10.1038/s41586-024-08351-7.

Xu, X.-F., Wang, Y.-C., Zong, L., and Wang, X.-L. (2019). miR-151-5p modulates APH1a expression to participate in contextual fear memory formation. RNA Biol. *16*, 282–294. https://doi.org/10.1080/15476286.2019.1572435.

Yale, A.R., Kim, E., Gutierrez, B., Hanamoto, J.N., Lav, N.S., Nourse, J.L., Salvatus, M., Hunt, R.F., Monuki, E.S., and Flanagan, L.A. (2023). Regulation of neural stem cell differentiation and brain development by MGAT5-mediated N-glycosylation. Stem Cell Rep. *18*, 1340–1354. https://doi.org/10.1016/j.stemcr.2023.04.007.

Yang, G., Zhou, R., Guo, X., Yan, C., Lei, J., and Shi, Y. (2021). Structural basis of γ-secretase inhibition and modulation by small molecule drugs. Cell *184*, 521–533.e14. https://doi.org/10.1016/j.cell.2020.11.049.

Yang, G., Zhou, R., Zhou, Q., Guo, X., Yan, C., Ke, M., Lei, J., and Shi, Y. (2019). Structural basis of notch recognition by human γ-secretase. Nature *565*, 192–197. https://doi.org/10.1038/s41586-018-0813-8.

Yapijakis, C. (2020). In Regulatory role of MicroRNAs in brain development and function, P. Vlamos, ed. (Springer International Publishing), pp. 237–247.

Zhou, J., Liu, G., Zhang, X., Wu, C., Ma, M., Wu, J., Hou, L., Yin, B., Qiang, B., Shu, P., and Peng, X. (2022). Comparison of the Spatiotemporal Expression Patterns of Three Cre Lines, Emx1IRES-Cre, D6-Cre and hGFAP-Cre, Commonly Used in Neocortical Development Research. Cereb. Cortex *32*, 1668–1681. https://doi.org/10.1093/cercor/bhab305.

Zhou, R., Yang, G., Guo, X., Zhou, Q., Lei, J., and Shi, Y. (2019). Recognition of the amyloid precursor protein by human γ-secretase. Science *363*, eaaw0930. https://doi.org/10.1126/science.aaw0930.

STAR★METHODS

KEY RESOURCES TABLE

REAGENT or RESOURCE	SOURCE	IDENTIFIER
**Antibodies**		
Anti-TBR1 antibody (rabbit polyclonal)	Abcam	Cat# ab31940; RRID: AB_2200219
Anti-TBR2 antibody (rabbit polyclonal)	Abcam	Cat# ab23345; RRID: AB_778267
Anti-PAX6 antibody (rabbit monoclonal)	Abcam	Cat# ab195045; RRID: AB_2750924
Anti-NEUROD2 antibody (rabbit polyclonal)	Abcam	Cat# ab104430; RRID: AB_10975628
Anti-CUX1 antibody	Oasis Biofarm	Cat# OB-PRT034-01; RRID:AB_2938851
Anti-CTIP2 antibody	Oasis Biofarm	Cat# OB-PRB025-01; RRID:AB_2938879
Anti-TLE4 antibody (mouse monoclonal)	Santa Cruz Biotechnology	Cat# sc-365406; RRID: AB_10841582
Anti-SOX5 antibody (rabbit polyclonal)	Abcam	Cat# ab94396; RRID: AB_10859923
Anti-SOX2 antibody (rabbit monoclonal)	Abcam	Cat# ab97959; RRID: AB_2341193
Anti-MAP2 antibody (mouse monoclonal)	Abcam	Cat# ab11267; RRID: AB_297885
Anti-GFAP antibody (rabbit polyclonal)	Abcam	Cat# ab7260; RRID: AB_305808
Anti-BrdU antibody (rat monoclonal)	Abcam	Cat# ab6326; RRID: AB_305426
Anti-KI67 antibody (rabbit monoclonal)	Abcam	Cat# ab15580; RRID: AB_443209
Anti-APH1A antibody (rabbit polyclonal)	Thermo Fisher Scientific	Cat# PA1-2010; RRID: AB_2227105
Anti-NICD antibody (rabbit monoclonal)	Cell Signaling Technology	Cat# 4147S; RRID: AB_2153348
Anti-mCherry antibody (rabbit polyclonal)	Thermo Fisher Scientific	Cat# M11217; RRID: AB_2536611
Anti-ALDH1L1 antibody (rabbit polyclonal)	Oasis Biofarm	Cat# OB-PRB001-01; RRID:AB_2934257
Anti-β-ACTIN antibody (mouse monoclonal)	Sigma-Aldrich	Cat# A5441; RRID: AB_476744
**Bacterial and virus strains**		
Escherichia coli DH5α chemically competent cells	This paper (lab-prepared)	Genotype: F– φ80dlacZΔM15 deoR Δ(lacZYA-argF)U169 recA1 endA1 hsdR17(rK– mK+) supE44 thi-1 gyrA96 relA1
**Chemicals, peptides, and recombinant proteins**		
Paraformaldehyde (PFA)	Sigma-Aldrich	Cat# P6148
BrdU (5-Bromo-2′-deoxyuridine)	MedChemExpress	Cat# HY-15910
EdU (5-ethynyl-2′-deoxyuridine)	Thermo Fisher Scientific	Cat# A10044
Recombinant human EGF	PeproTech	Cat# AF-100-15
Recombinant human bFGF	PeproTech	Cat# 100-18B
TRIzol Reagent	Thermo Fisher Scientific	Cat# 15596018
DAPI (4′,6-diamidino-2-phenylindole)	Sigma-Aldrich	Cat# D9542
Gibco™ BASIC DMEM, High Glucose, Pyruvate	Thermo Fisher Scientific	Cat# C11995500BT
DMEM/F-12, HEPES	Thermo Fisher Scientific	Cat# 11330032

(Continued on next page)

Continued

REAGENT or RESOURCE	SOURCE	IDENTIFIER
B-27™ Supplement (50X), serum free	Thermo Fisher Scientific	Cat# 17504044
Penicillin-Streptomycin (100X)	Thermo Fisher Scientific	Cat# 15140122
Lipofectamine™ 3000	Thermo Fisher Scientific	Cat# L3000075
**Critical commercial assays**		
Click-iT™ EdU Cell Proliferation Kit for Imaging, Alexa Fluor™ 647 dye	Thermo Fisher Scientific	Cat# C10340
Phanta UniFi	Vazyme	Cat# P516/526-01
HiScript III RT SuperMix for qPCR (+gDNA wiper)	Vazyme	Cat# R323-01
TB Green Premix Ex Taq II (Tli RNase H Plus)	TaKaRa	Cat# RR820A
Plasmid Miniprep Kit	TIANGEN	Cat# DP103
Universal DNA Purification and Recovery Kit	TIANGEN	Cat# DP214
EndoFree Plasmid Maxi Kit (10)	QIAGEN	Cat# 12362
Dual-Glo Luciferase Assay System	Promega	Cat# E2920
**Deposited data**		
RNA-seq Raw and analyzed data	This paper	GEO:GSE327779
**Experimental models: Cell lines**		
HEK293T	ATCC	RRID: CVCL_0063
HeLa	ATCC	RRID: CVCL_0030
N1E-115	ATCC	RRID: CVCL_0451
**Experimental models: Organisms/strains**		
Mus musculus C57BL/6J wild-type mice	Institute of Laboratory Animal Science, Chinese Academy of Medical Sciences	Strain: C57BL/6J
Mus musculus Emx1^{IRES}-Cre mice	Gift from Prof. Zhengang Yang (Fudan University)	JAX: 005628
Mus musculus miR-151 fL/fL mice	Biocytogen (Beijing, China)	Conditional allele: miR-151-loxP
Mus musculus ICR pregnant mice	Peking University Health Science Center	Timed pregnant (plug-checked; ±0.5 days)
Mus musculus Emx1-miR-151 conditional knockout mice	This paper	Generated by crossing miR-151 ^fl/fl mice with Emx1^{IRES}-Cre mice
Primary neural stem cells (mouse)	This paper	Isolated from E14.5 mouse cortex
**Oligonucleotides**		
miR-151–5p mimics	This paper	UCGAGGAGCUCACAGUCUAGU
miR-151–5p mimics NC	This paper	UUGUACUACACAAAAGUACUG
U6-RT	This paper	GTCGTATCCAGTGCAGGGTCCGAGGTATTCGC ACTGGATACGACAAAAATATG
miR-151–5p RT	This paper	GTCGTATCCAGTGCAGGGTCCGAGGTATTCGC ACTGGATACGACACTAGA
U6-rltm-F	This paper	GCGCGTCGTGAAGCGTTC
miR-151–5p rltm-F	This paper	GCCCCTCGAGGAGCTCAC

(*Continued on next page*)

Continued		
REAGENT or RESOURCE	SOURCE	IDENTIFIER
miR-rltm-R	This paper	GTGCAGGGTCCGAGGT
**Recombinant DNA**		
pCIG-APH1A	This paper	N/A
pCIG-miR-151	This paper	N/A
miR-151–5p Sponge	This paper	N/A
PB-pCIG-miR-151	This paper	N/A
CBFRE-mCherry	This paper	N/A
**Software and algorithms**		
LAS X	Leica	LAS_X_4.7.0
FV10-ASW Viewer	Olympus	FV10-ASW Viewer software (Ver.4.2b)

EXPERIMENTAL MODEL AND STUDY PARTICIPANT DETAILS

Experimental animals

Mice carrying miR-151 flanked by the *loxP* allele were generated by Biocytogen Co., Ltd. (Beijing) and after mated with Emx1-IRES-Cre (Jackson Laboratories, stock number 005628) mice to generate miR-151 cko mice, which were in C57BL/6J background(Zhou et al., 2022). The mice were given free access to food and water. All animal experiments were conducted according to protocols approved by the Institutional Animal Care and Use Committee at the Academy of Medical Sciences and Peking Union Medical College (ACUC-A01-2025-010). The noon of the day when the vaginal plug was found was counted as embryo (E) day 0.5.

Bacterial strains and culture conditions

Chemically competent Escherichia coli DH5α cells were prepared in-house in our laboratory and used for plasmid amplification and molecular cloning. The genotype of the DH5α strain was F− φ80dlacZΔM15 deoR Δ(lacZYA-argF)U169 recA1 endA1 hsdR17(rK− mK+) supE44 thi-1 gyrA96 relA1. Cells were maintained and cultured in LB medium at 37°C under standard bacterial growth conditions.

Cell culture

HEK293T, N1E-115 and HeLa cells were cultured in Dulbecco's modified Eagle's medium (DMEM) with 10% fetal bovine serum and at 37°C with 5% CO_2 in a humid incubator. Lipofectamine 3000 was used for plasmid transfection according to the manufacturer's instructions. HEK293T, HeLa, and N1E-115 cells were authenticated by source information and routinely tested negative for mycoplasma contamination.

Mouse NSCs from E14.5 and E16.5 mice were obtained by dissecting the lateral ventricles, followed by digestion into a single-cell suspension with Accutase (Sigma). NSCs were maintained in DMEM/F12 proliferation medium supplemented with 2% B27 supplement, 20 ng/mL EGF, 20 ng/mL bFGF, 1% GlutaMAX supplement and 0.2% BSA. After culture *in vitro* for three generations, NSCs were subjected to Western blotting, RT−qPCR and RNA−seq. NSCs were prepared freshly and used for experiments without long-term banking.

METHOD DETAILS

Tissue section

The pregnant mice were intraperitoneally anesthetized with 0.7% w/v pentobarbital sodium (0.01mL/g body weight). Embryonic mouse brains were then dissected out in cold PBS. After being anesthetized with the same agent, P3 mice were subjected to transcardial perfusion with 4% PFA-PBS. Both embryonic and P3 brain samples were fixed in 4% PFA for 24 h, followed by immersion in 25% sucrose solution for another 24 h for cryoprotection. The brains were subsequently embedded in O.C.T. (SAKURA) and stored at −80°C. They were then cut into 16 μm thick sections using a Leica CM1950 cryostat.

Immunofluorescence

The tissue sections were desiccated at 50°C for 30 min and then washed in 1×PBS for 5 min. For heat-mediated antigen retrieval, the sections were incubated with 10 mM sodium citrate buffer (pH 6.0) at 95°C for 20 min, followed by natural cooling. For BrdU staining, the sections were treated with 2N HCl for 10 min at 30°C and subsequently for 20 min at room temperature. The sections were subsequently incubated with 5% sheep serum (1×PBS, 0.3% Triton X-100) for 1 h at room temperature. The tissue sections were incubated overnight at 4°C with primary antibodies, prepared in buffer (1×PBS, 0.3% Triton X-100). The primary antibody mixture was removed, and the sections were washed three times in PBS for 5 min each. The secondary antibodies were diluted with buffer solution at a ratio of 1:800 and incubated with the tissue sections for 2 h at room temperature in the dark. After three rinses in PBS, the nuclei were stained with 4′,6-diamidino-2-phenylindole (DAPI) and then mounted. The tissue slices were observed and photographed with a Leica system (Leica Stellaris 5 Confocal Microscope).

5-Ethynyl-2′-deoxyuridine (EdU) staining

Cell proliferation was assessed using the Click-iT Plus EdU Cell Proliferation Kit for Imaging according to the manufacturer's protocols. In brief, following standard immunofluorescence staining, the sections were treated with the EdU working solution for 30 min at room temperature in the dark prior to mounting.

In utero electroporation

In utero electroporation (IUE) was performed as previously described (Shu et al., 2019b). Pregnant mice were anesthetized with 0.7% w/v pentobarbital sodium (0.01mL/g body weight). E13.5 embryonic brains received five 30 V pulses (50ms on/950ms interval) via 7-mm platinum electrodes using a BTX-ECM830 electroporator (Harvard Apparatus). All plasmids used for IUE were based on the pCIG vector, which contains IRES and EGFP elements, allowing co-translation with the target protein, all plasmids were electroporated at 2.5 μg/μL(Megason and McMahon, 2002).

Neurosphere formation assay for NSCs

To evaluate the self-renewal capacity of NSCs, we performed a neurosphere formation assay based on sphere-forming efficiency. NSCs were first dissociated into a single-cell suspension using Accutase. Cell density was determined using an automated cell counter, and cells were seeded at a standardized density of 100,000 cells per well in 24-well plates. Following 3 days of culture, images were acquired from ten randomly selected fields of view per well. Neurospheres were quantified and stratified based on their radii (0–50 μm, 50–100 μm, and >100 μm). The number and size distribution of these neurospheres were used to calculate the sphere-forming efficiency, serving as a functional readout of NSC stemness.

Western blot

NSCs and forebrains were swiftly isolated and lysed in TNTE buffer (50 mM Tris-HCl, 150 mM NaCl, 1 mM EDTA, 1 mM Na_3VO_4, 25 mM NaF, 10 mM $Na_4P_2O_7 \cdot 10H_2O$, 0.5% Triton X-100 and protease inhibitors). The lysates were incubated for 30 min on ice and centrifuged at 12,000 rpm for 30 min at 4°C. Proteins were separated on 10% SDS−PAGE gels and transferred to nitrocellulose membranes. Prior to incubation with primary antibodies overnight, the membranes were blocked with 5% nonfat milk dissolved in TBS-Tween 20 (0.05%) for 1 h at room temperature.

RT−qPCR

Total RNA from NSCs of control and miR-151 cko mice was isolated using TRIzol reagent (Invitrogen). cDNA was synthesized using a Reverse Transcriptase Kit. Quantitative RT−PCR was conducted with an SYBR Green-containing kit (TaKaRa). The primer sequences could be found in Table S1, with *Gapdh* and *U6* serving as the internal control.

Luciferase assays

HEK293T cells in a 24-well plate were cotransfected with the reporter plasmid (psiCHECK-2 Vector, Promega) and miR-151–5p mimics. The cells were collected after 72 h, and the Dual-Luciferase Reporter Assay System from Promega was used to measure the activity levels of both firefly and Renilla luciferase within the same sample. The activity of each reporter was evaluated over the course of three separate experiments, with each experiment consisting of three replicates.

Fear conditioning behavioral experiment

Habituation (Day 1): Animals are briefly exposed to the testing apparatus without any stimuli to reduce novelty stress.

Training (Day 2): Animal is placed in the conditioning chamber (Context A). After a baseline period (180 s), a neutral conditioned stimulus (CS; tone, 30 s, 80 dB) is presented. The CS terminates with the onset of an aversive unconditioned stimulus (US; mild footshock, 0.8 mA, 1 s). This CS-US pairing is repeated 5 times with 60 s inter-trial intervals (ITIs). Animal remains in the chamber briefly after the last shock (60 s) before returning to home cage.

Context Test (Day 3): 24 h post-conditioning, the animal is placed back into the original conditioning chamber (Context A). No CS or US is presented.

Cued Test (Day 4): 24 h after the context test, the animal is placed into a novel, altered chamber (Context B) - different shape, smell, lighting. After a baseline period (180 s) to assess generalized fear in the new context, the CS (tone) is presented alone without US. Freezing is scored during the pre-CS baseline and during the CS presentation. Increased freezing specifically during the CS indicates cue-specific fear conditioning.

RNA seq

Total RNA from E14.5 NSCs from both control and miR-151 cko mice was isolated using TRIzol reagent (Invitrogen). RNA-seq libraries were generated and sequenced by CapitalBio Technology (Beijing, China). The sequencing quality was assessed with FastQC (v0.11.5), and low-quality data were filtered out via NGSQC (v2.3.3). The clean reads were then aligned to the mouse UCSC mm10 genome using HISAT2 with default parameters. The processed reads from each sample were aligned against the reference genome via HISAT2. DESeq2 was used to identify differentially expressed genes between samples. Gene set enrichment analysis (GSEA) in this study was performed using clusterProfiler. To identify significantly enriched Gene Ontology (GO) terms (FDR <0.05), the Database for Annotation, Visualization and Integrated Discovery was used.

QUANTIFICATION AND STATISTICAL ANALYSIS

Image analysis, quantification and statistical analysis

Images were captured to cover each coronal section with a 10x or 40× objective by Leica Stellaris 5 confocal microscope and compared with equivalent sections in littermate counterparts. Brightness and contrast were adjusted using Photoshop where needed. For each separate experiment, three or more embryos were used for qualitative analyses ($n \geq 3$). Statistical analyses were performed using GraphPad Prism 9.5.0. Results are presented as mean ± SEM. Unpaired two-tailed *t*-tests were used to compare two datasets. For each comparison, numbers from at least 3 individually samples were averaged.

Stem Cell Reports, Volume *21*

Supplemental Information

miR-151-5p regulates neural stem cell fate by targeting APH1A to modulate Notch signaling gradients

Xinrun Wang, Li Li, Zhuo Chen, Yi Zeng, Pengcheng Shu, Lin Hou, Bin Yin, Wei Liu, and Xiaozhong Peng

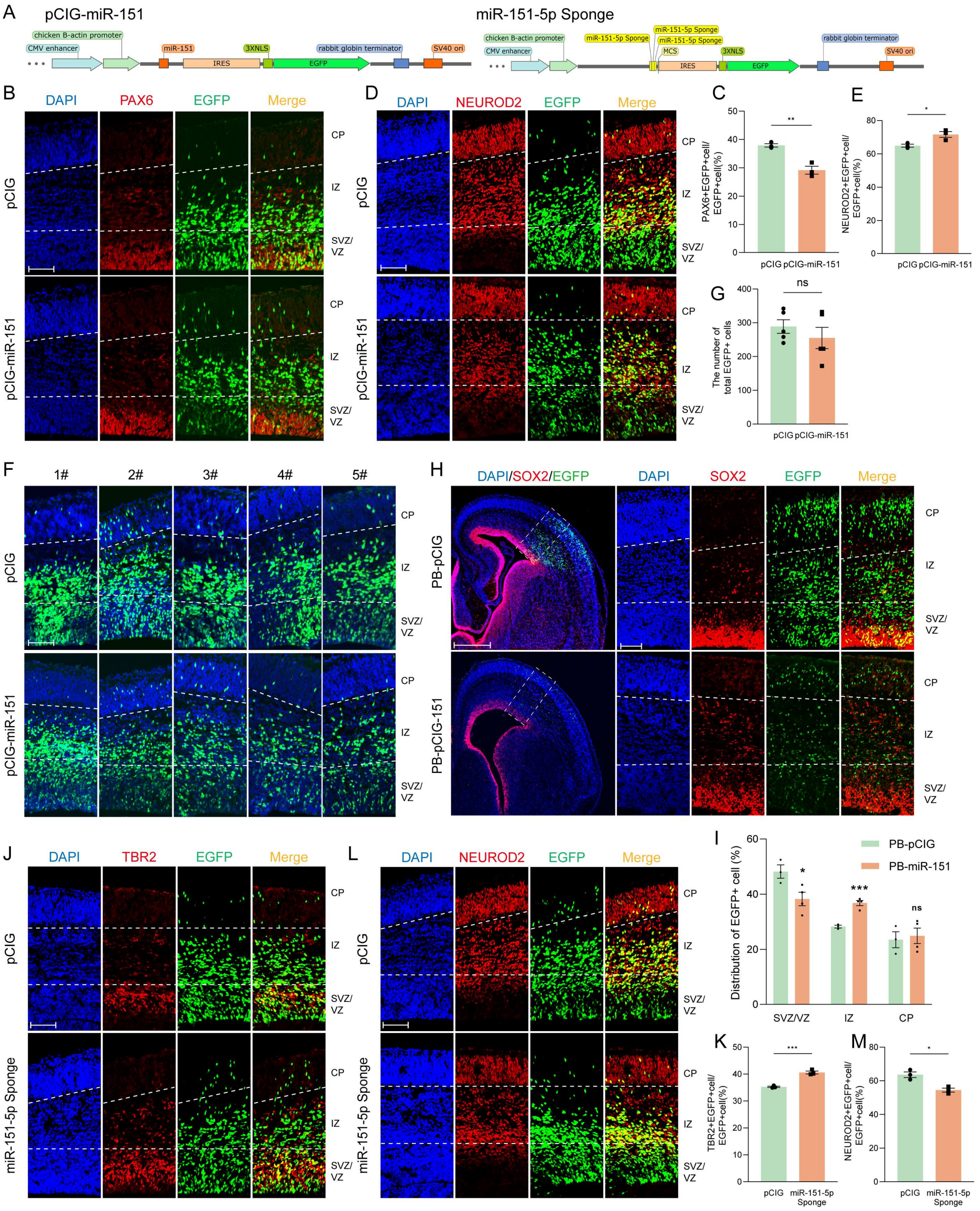

Supplementary Figure 1. miR-151-5p influences the outward migration of NSCs.

(A) Schematic diagram showing pCIG-miR-151 (overexpression) and miR-151-5p Sponge (knockdown) plasmid.

(B) Immunostaining of E15.5 brain sections electroporated with control (pCIG) or OE (pCIG-miR-151) on E13.5. White-dotted rectangles mark comparable regions for magnification.

(C) Quantification of the ratio of $PAX6^{+}EGFP^{+}$cells in all $EGFP^{+}$ cells.

(D) Immunostaining of E15.5 brain sections electroporated with control (pCIG) or OE (pCIG-miR-151) on E13.5. White-dotted rectangles mark comparable regions for magnification.

(E) Quantification of the ratio of $NEUROD2^{+}EGFP^{+}$cells in all $EGFP^{+}$ cells.

(F) Representative images from five independent experiments, showing E15.5 brain sections that were electroporated on E13.5 with the control (pCIG) or OE (pCIG-miR-151).

(G) Quantitative analysis of the number of total $EGFP^{+}$ cells, n=5 biological replicates.

(H) Immunostaining of E16.5 brain sections electroporated with control (PB-pCIG) or miR-151-OE plasmid on E13.5. White-dotted rectangles mark comparable regions for magnification.

(I) Quantitative analysis of the distribution of $EGFP^{+}$ cells after dividing the neocortex into 3 parts (SVZ/VZ, IZ, CP) (n=3 for the control group, n=4 for the miR-151 OE group).

(J) Immunostaining of E15.5 brain sections electroporated with control (pCIG) or Down (miR-151-5p Sponge) on E13.5. White-dotted rectangles mark comparable regions for magnification.

(K) Quantification of the ratio of $TBR2^{+}EGFP^{+}$cells in all $EGFP^{+}$ cells.

(L) Immunostaining of E15.5 brain sections electroporated with control (pCIG) or Down (miR-151-5p Sponge) on E13.5. White-dotted rectangles mark comparable regions for magnification.

(M) Quantification of the ratio of $NEUROD2^{+}EGFP^{+}$cells in all $EGFP^{+}$ cells.

Scale bars for the 10x images, 500 μm; 40x images, 100 μm. Data are presented as mean ± SEM. Individual data points represent independent biological replicates. Statistical analysis was performed by unpaired 2-tailed Student's *t* test; ns, not significant; * $p < 0.05$; *** $p < 0.001$.

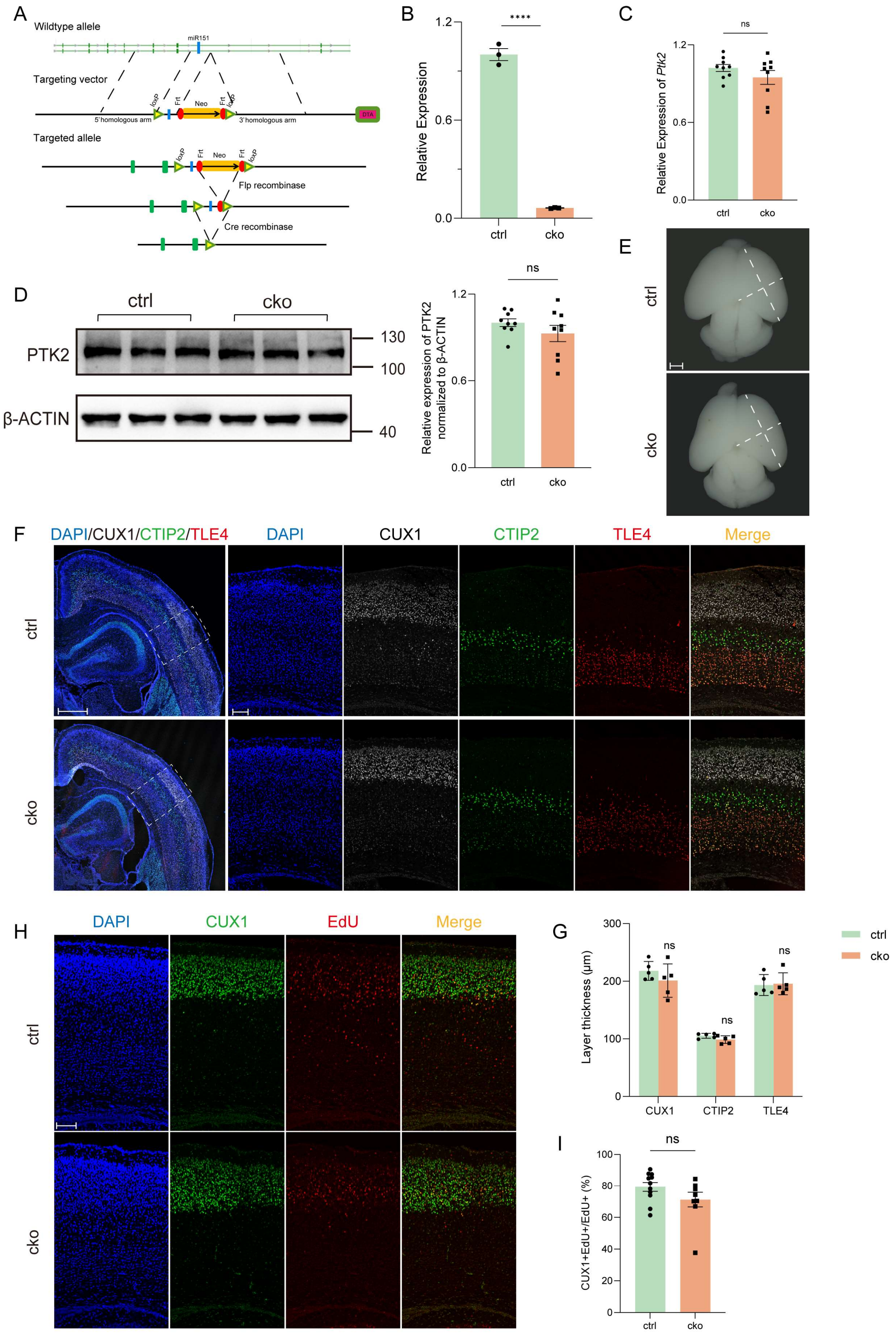

Supplementary Figure 2. Knockout miR-151-5p does not affect the construct of the cerebral cortex in postnatal mice.

(A) Schematic diagram of the construction strategy of conditional knockout miR-151 mouse, loxP was inserted on the intron sequence between exons 22 and 23 of *Ptk2* gene.

(B) RT-qPCR measurement of miR-151-5p expression level in E14.5 miR-151 cko and ctrl NSC, n=3 biological replicates.

(C) Measurement of *Ptk2* mRNA expression in NSCs derived from E14.5 ctrl and miR-151-cko littermate mice. n=3 biological replicates.

(D) Western blot of PTK2 in NSCs from E14.5 miR-151 cko and ctrl littermate mice. β-ACTIN is used as a loading control. ImageJ is used to analyze the gray scale of signals.

(E) Brain dissection revealed that loss of miR-151 did not affect the dorsal forebrain at P3 mouse. Scale bar, 1mm.

(F) Immunostaining of P3 brain sections derived from miR-151 cko and ctrl littermate mice. Sections were stained with DAPI and various markers (CUX1, CTIP2, TLE4). White-dotted rectangles mark comparable regions for magnification.

(G) Quantitative analysis of the thickness of different cortical layers, n=5 biological replicates.

(H) Immunostaining of P3 brain sections derived from miR-151 cko and ctrl littermate mice. Sections were stained with DAPI, upper-layer marker CUX1 and EdU labeling.

(I) Quantitative analysis of the ratio of $CUX1^+EdU^+$ cells in all EdU^+ cells, n=7 biological replicates.

Scale bars for the 10x images, 500 μm; 40x images, 100 μm. Data are presented as mean ± SEM. Individual data points represent independent biological replicates. Statistical analysis was performed by unpaired 2-tailed Student's *t* test; ns. not significant; **** $p < 0.0001$.

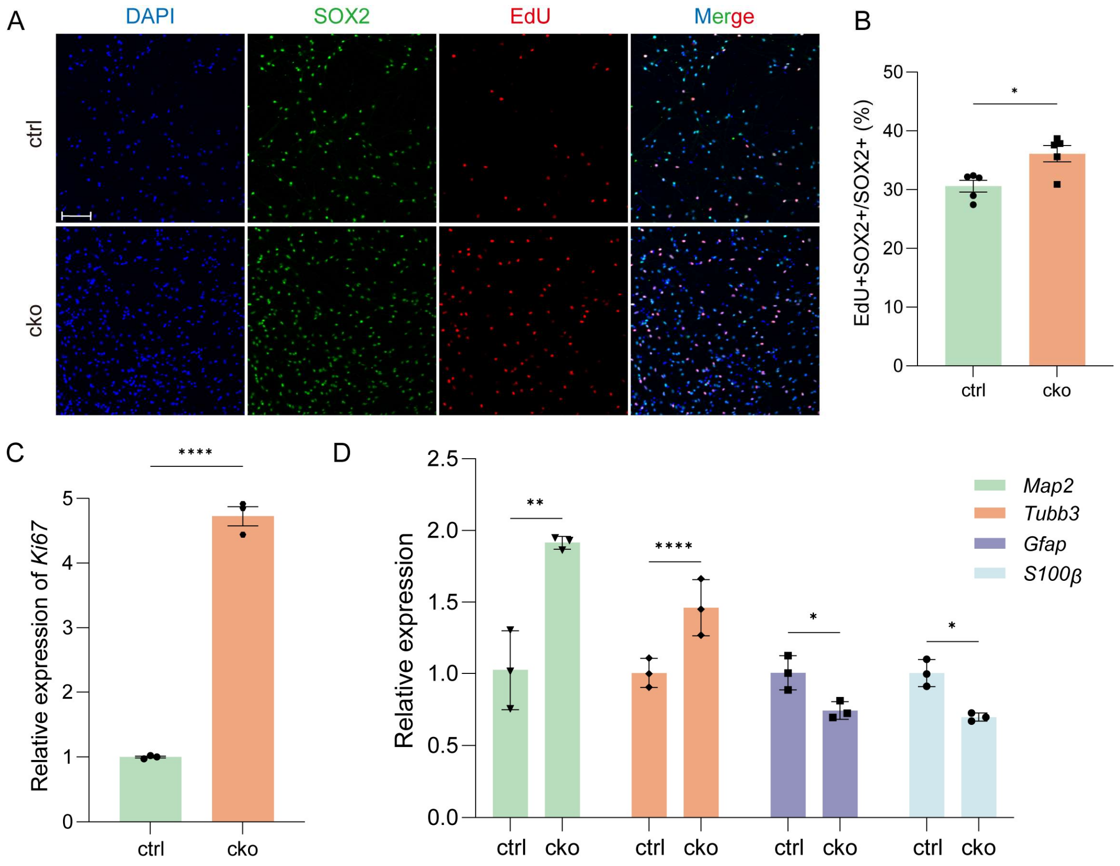

Supplementary Figure 3. Knockout miR-151 changes the proliferative ability and differentiation potential of NSC.

(A) Detection of NSC proliferative ability from E16.5 miR-151 cko and ctrl littermate mice by immunostaining. Scale bar, 100 μm.

(B) Quantitative analysis of the ratio of $EdU^{+}SOX2^{+}$ cells in all $SOX2^{+}$ cells, n=5 biological replicates.

(C) Measurement of *Ki67* mRNA expression in NSCs derived from E14.5 ctrl and miR-151-cko littermate mice. n=3 biological replicates.

(D) Measurements of neuron markers (*Map2*, *Tubb3*) and astrocyte markers (*Gfap*, *S100β*) mRNA expression in NSCs derived from E16.5 ctrl and miR-151-cko littermate mice. n=3 biological replicates.

Data are presented as mean ± SEM. Individual data points represent independent biological

replicates. Statistical analysis was performed by unpaired 2-tailed Student's *t* test; * $p < 0.05$; ** $p < 0.01$; *** $p < 0.001$; **** $p < 0.0001$.

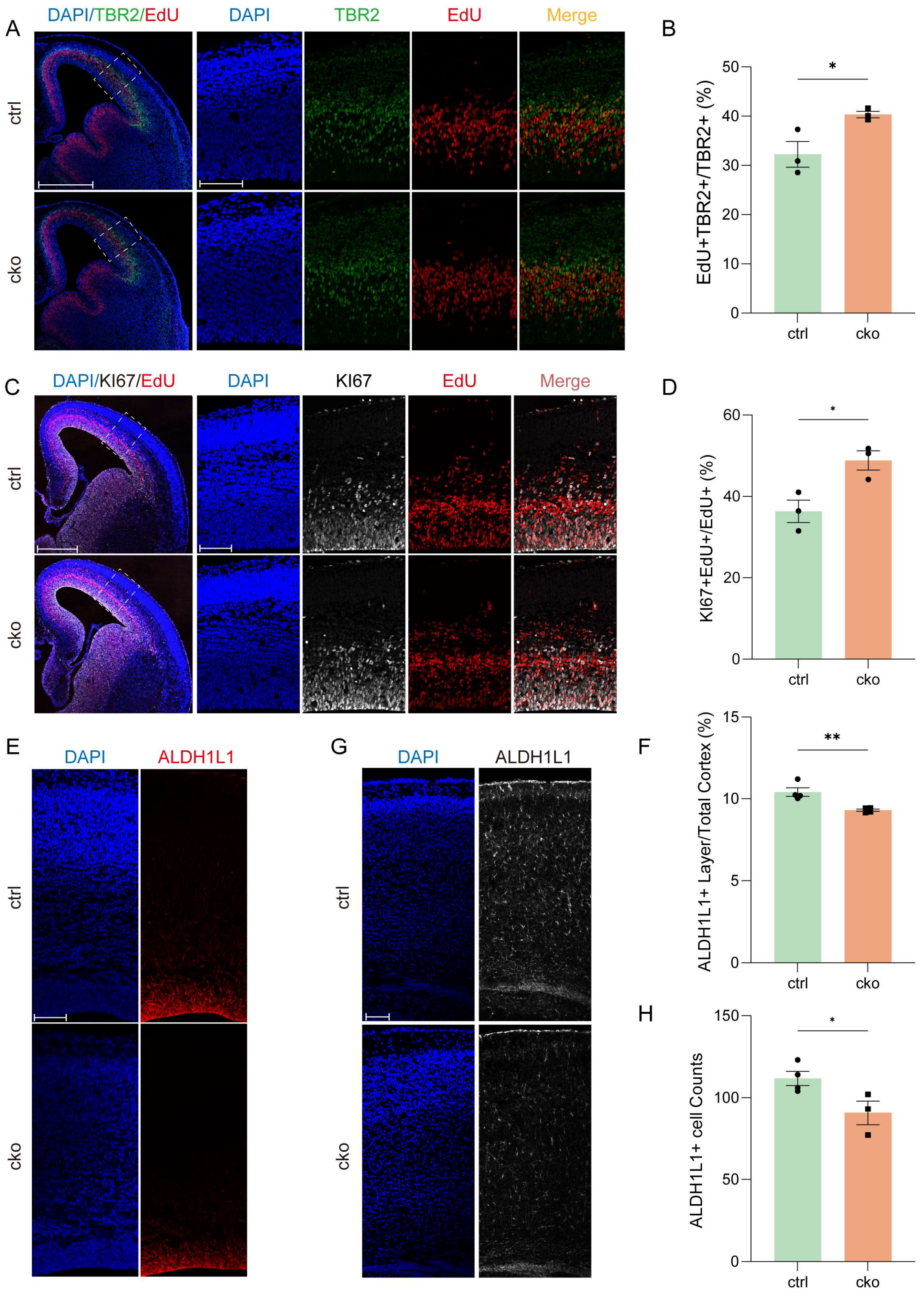

Supplementary Figure 4. Knockout miR-151 decreases ALDH1L1$^+$ cells in gliogenesis.

(A) Immunostaining of E13.5 brain sections derived from miR-151 cko and ctrl littermate mice. White-dotted rectangles mark comparable regions for magnification.

(B) Quantitative analysis of the ratio of EdU$^+$TBR2$^+$ cells in all TBR2$^+$ cells, n=3 biological

replicates.

(C) Immunostaining of E15.5 coronal sections derived from miR-151 cko and ctrl littermate mice. White-dotted rectangles mark comparable regions for magnification.

(D) Quantification of the ratio of $KI67^{+}EdU^{+}$ cells in all EdU^{+} cells.

(E) Immunostaining of E18.5 coronal sections derived from miR-151 cko and ctrl littermate mice. Sections were stained with DAPI staining and astrocyte marker ALDH1L1.

(F) Quantification of the ratio of $ALDH1L1^{+}$ layer in total cortex.

(G) Immunostaining of P3 coronal sections derived from miR-151 cko and ctrl littermate mice. Sections were stained with DAPI staining and astrocyte marker ALDH1L1.

(H) Quantification of the number of $ALDH1L1^{+}$ cells.

Scale bars for the 10x images, 500 μm; 40x images, 100 μm. Data are presented as mean ± SEM. Individual data points represent independent biological replicates. Statistical analysis was performed by unpaired 2-tailed Student's *t* test, n=3 biological replicates; $*p < 0.05$; $**p < 0.01$.

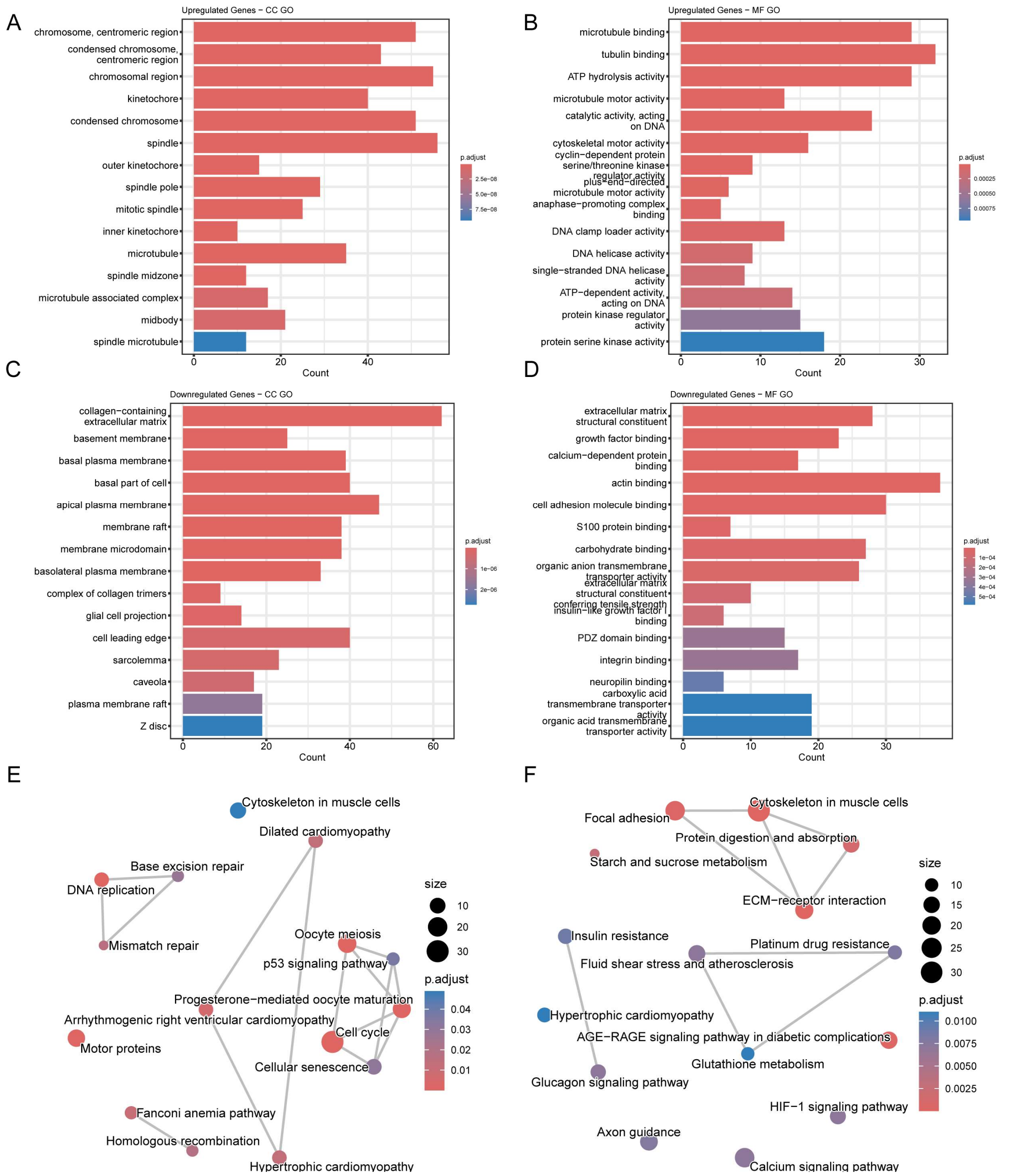

Supplementary Figure 5. GO enrichment results and KEGG-Pathway enrichment results in E14.5 miR-151 cko and ctrl NSCs.

(A) The GO enrichment results of cellular components were obtained by using up-regulated genes.

(B) The GO enrichment results of molecular functions were obtained by using up-regulated genes.

(C) The GO enrichment results of cellular components were obtained by using down-regulated genes.

(D) The GO enrichment results of molecular functions were obtained by using down-regulated genes.

(E) The enrichment map is used to visualize the correlations between various pathways in the results of KEGG-Pathway enrichment analysis by using up-regulated genes.

(F) The enrichment map is used to visualize the correlations between various pathways in the results of KEGG-Pathway enrichment analysis by using down-regulated genes.

All results were analyzed by using clusterProfiler.
